# Hyperglycemia enhances pancreatic cancer progression accompanied by elevations in phosphorylated STAT3 and MYC levels

**DOI:** 10.1371/journal.pone.0235573

**Published:** 2020-07-01

**Authors:** Katsuhiko Sato, Hayato Hikita, Yuta Myojin, Kenji Fukumoto, Kazuhiro Murai, Sadatsugu Sakane, Takeshi Tamura, Takuo Yamai, Yasutoshi Nozaki, Teppei Yoshioka, Takahiro Kodama, Minoru Shigekawa, Ryotaro Sakamori, Tomohide Tatsumi, Tetsuo Takehara

**Affiliations:** Department of Gastroenterology and Hepatology, Osaka University Graduate School of Medicine, Osaka, Japan; Nihon University School of Medicine, JAPAN

## Abstract

Diabetes mellitus is a well-known risk factor for pancreatic cancer. We focused on hyperglycemia, a main feature of diabetes mellitus, and uncovered its effect on precancerous pancreatic intraepithelial neoplasia (PanIN) progression. In vivo induction of hyperglycemia with 100 mg/kg streptozotocin in *Kras*^*LSL G12D*^ *Pdx1Cre* (KP) mice promoted the PanIN formation and progression. Preconditioning with a high- or low-glucose medium for 28 days showed that a high-glucose environment increased cell viability and sphere formation in PANC-1, a Kras-mutant human pancreatic ductal adenocarcinoma cell line, and mPKC1, a Kras-mutant murine pancreatic cancer cell line. In contrast, no changes were observed in BxPC3, a Kras-wild-type human pancreatic cancer cell line. Orthotopic injection of mPKC1 into the pancreatic tails of BL6/J mice showed that cells maintained in high-glucose medium grew into larger tumors than did those maintained in low-glucose medium. Hyperglycemia strengthened the STAT3 phosphorylation, which was accompanied by elevated MYC expression in Kras-mutant cells. Immunohistochemistry showed stronger phosphorylated STAT3 (pSTAT3) and MYC staining in PanINs from diabetic KP mice than in those from euglycemic counterparts. STAT3 inhibition with 1 μM STAT3 inhibitor STATTIC in Kras-mutant pancreatic cell lines blocked the cell viability- and sphere formation-enhancing effects of the hyperglycemic environment and reversed the elevated pSTAT3 and MYC expression. MYC knockdown did not affect cell viability but did reduce sphere formation. No decrease in pSTAT3 expression was observed upon siMYC treatment. In conclusion, hyperglycemia, on a Kras-mutant background, aggravates the PanIN progression, which is accompanied by elevated pSTAT3 and MYC expression.

## Introduction

Progress in cancer research has not led to significant improvements in the survival of patients with pancreatic cancer. The five-year survival rate of patients remains as low as 6.9% [1], which is not only due to the malignant nature of this cancer but also to difficulties in its early detection [2, 3]. Diabetes mellitus is a well-known risk factor for pancreatic cancer. Up to 25.9% of pancreatic cancer patients have diabetes, and in turn, diabetic patients have a two-fold higher risk of pancreatic cancer than nondiabetic patients [4, 5]. Similar to diabetes mellitus, obesity [6, 7] and chronic pancreatitis [8] are known clinical risk factors for pancreatic cancer. Kras mutations are found in more than 90% of patients with pancreatic cancer [9]. It has been shown using genetically engineered oncogenic Kras mice that a high-fat diet and pancreatitis accelerated pancreatic intraepithelial neoplasia (PanIN) progression [10–12]. However, no study has focused on diabetes and its effect on PanIN.

Hyperglycemia is one of the most important aspects of diabetes mellitus. Type 1 diabetes, which is characterized by hyperglycemia and low blood insulin levels, is a pancreatic cancer risk factor [13]. In patients with a diabetes duration of 5 years or more, hyperglycemia is an independent risk factor for pancreatic cancer [14]. Hyperglycemia has been reported to precede a pancreatic cancer diagnosis by 30–36 months, illustrating the possibility that this condition is a tumor enhancer [15]. Pancreatic cancer may be responsible for new-onset diabetes, but in the long run, prolonged diabetes contributes to cancer progression [2, 16, 17]. These clinical reports have shown that hyperglycemia may be an important risk factor for pancreatic cancer. However, how hyperglycemia enhances pancreatic cancer progression remains a major question.

The aims of our study were to clarify whether hyperglycemia promotes pancreatic cancer progression and to elucidate the underlying mechanism. Here, we show that hyperglycemia aggravates precancerous PanIN progression in genetically engineered mouse models of pancreatic cancer. Hyperglycemia did not activate ERK, the major Kras effector, but strengthened the phosphorylation of STAT3 and the expression of MYC. In in vitro experiments, high-glucose preconditioning enhanced STAT3 phosphorylation and MYC expression in Kras-mutant pancreatic ductal cells, which was similar to the enhancements observed in diabetic oncogenic Kras mouse models. STAT3 inhibition decreased MYC expression, showing the existence of the STAT3-MYC pathway in pancreatic ductal cell lines. On a Kras-mutant background, hyperglycemia may contribute to pancreatic cancer progression via STAT3 phosphorylation and elevated MYC expression.

## Materials and methods

### Animal studies

*Pdx1-Cre* transgenic mice and *Kras*^*LSL G12D*^ knock-in mice were both obtained from the Mouse Models of Human Cancer Consortium (National Cancer Institute, Frederick, Bethesda, Maryland). *Trp53*^*fl/fl*^ mice and *EYFP*^*Tg/Tg*^ mice were purchased from the Jackson Laboratory (Bar Harbor, ME, USA). *ElaCre* mice were obtained from the Center for Animal Resources and Development (Kumamoto University, Kumamoto, Japan). *Pdx1Cre* mice and *Kras*^*LSL G12D*^ mice were interbred to obtain *Kras*^*LSL G12D*^ *Pdx1Cre* (KP) mice and *Pdx1Cre* mice. All the purchased and bred mice were on the BL6/J background. Seven days after birth, mice were administered an intraperitoneal injection of 100 mg/kg streptozotocin (STZ) (S0130, Sigma, MO, USA), which was diluted in citrate buffer. Mice with blood sugar levels over 300 mg/dL at 8 weeks after birth were considered diabetic. Control mice were administered an intraperitoneal injection of citrate buffer only. Mice were sacrificed using an intraperitoneal injection of 200 mg/kg pentobarbital at 12 weeks of age, and their tissues were analyzed. Tissues were fixed with 4% paraformaldehyde or snap-frozen in liquid nitrogen. Animals were kept on a 12-hr/12-hr light/dark cycle and provided food and water *ad libitum*. All procedures performed in this study, including the use of primary cells and orthotopic models, were approved by the Committee of Experimental Animal Science of Osaka University and conducted within its regulations (Approval Number 30–015). All measures were taken to minimize animal suffering.

### Primary cell line

We established mPKC1, a murine primary pancreatic cancer cell line, as described previously [18]. A primary pancreatic tumor from an *ElaCre Kras*^*LSL G12D*^ *trp53*^*fl/fl*^ *EYFP*^*Tg/Tg*^ mouse was minced immediately after dissection and incubated with collagenase (1 mg/ml) in a shaker at 37°C for 20 min. Dissociated pieces of the tumor were incubated in 6-well plates containing Dulbecco’s modified eagle medium (DMEM) supplemented with 10% fetal bovine serum and 1% penicillin/streptomycin. The cells that grew from the pieces of the tumor were passaged to form a new murine pancreatic cancer cell line, mPKC1. Fluorescence microscopy was used to detect the pancreas-specific EYFP expression, which is induced by *ElaCre*, thus confirming that the cells were derived from the pancreas. The tumor formation by these cells was examined in an orthotopic pancreatic tumor model (S1A and S1B Fig).

### Orthotopic model

BL6/J mice were anesthetized with midazolam, butorphanol, and medetomidine prior to surgical resection, and all efforts were made to reduce animal suffering. A total of 1.0×10^5^ mPKC1 cells were suspended in Matrigel (356230, Corning, NY, USA) and injected into the tail of the mouse pancreas. The mice were sacrificed using an intraperitoneal injection of 200 mg/kg pentobarbital 14 days after orthotopic injection. The weights of dissected tumors were determined.

### Cell culture

The human pancreatic cancer cell lines PANC-1 (CRL-1469) and BxPC3 (CRL-1687) were obtained from ATCC (Manassas, VA, USA). The murine pancreatic cancer cell line mPKC1 was a primary cell line, which was cultured as described above. All the cell lines were used within 20 passages of purchase or establishment. Prior to preconditioning, all the cell lines were maintained in a high-glucose (25 mM) DMEM supplemented with 10% fetal bovine serum and 1% penicillin/streptomycin at 37°C in a humidified incubator with 5% CO_2_. For low- or high-glucose preconditioning, high-glucose-maintained cells were divided between low-glucose (5.5 mM) and high-glucose (25 mM) media with 5×10^5^ cells per dish. Cells from both low- and high-glucose media were then passaged once a week on the same day for 28 days, using the respective medium and plating 5×10^6^ cells per dish. The STAT3 inhibitor STATTIC (S7947, Sigma) was dissolved in DMSO and used at a concentration of 1 μM. The AKT inhibitor MK2206 2HCL (S1078, Sellek, TX, USA) was dissolved in DMSO and used at a concentration of 10 μM. All cell lines were tested for *Mycoplasma* within three months of use.

### WST-8 assay

Cells were maintained in the low- or high-glucose medium prior to experiments. A total of 2.0×10^3^ cells per well were placed in 96-well plates in 100 μL of DMEM with the low or high glucose concentration. Cell viability was evaluated by a WST-8 assay using a cell count reagent kit (07553, Nacalai Tesque, Kyoto, Japan). The absorbance was measured at 450 nm using a microplate reader (Thermo Fisher Scientific, MA, USA) according to the manufacturer’s instructions.

### BrDU cell proliferation assay

Cells were incubated in the low- or high-glucose medium before experiments. A total of 2.0×10^3^ cells per well were placed in 96-well plates in 100 μL of DMEM with the low or high glucose concentration. Cell proliferation was measured by cell proliferation ELISA using a BrDU (colorimetric) kit (11647229001, Sigma). The absorbance was measured at 370 nm using a microplate reader (Thermo Fisher Scientific).

### Annexin V assay

Cells were pretreated in the low- or high-glucose environment. A total of 1.0×10^5^ cells per well were seeded in 6-well plates. At 48 hr after seeding, annexin V-positive and propidium iodide (PI)-negative cells were counted using the following kits. An annexin V apoptosis detection kit FITC (88-8005-72, Thermo Fischer Scientific) was used for PANC-1 cells. To decrease interference with the EYFP fluorescence, an annexin V apoptosis detection kit APC (88-8007-72, Thermo Fisher Scientific) was used for mPKC1 cells. Cells were treated according to the manufacturer’s guidance and analyzed using a BD FACS Canto II flow cytometer (BD Biosciences, CA, USA).

### Sphere formation assay

Cells were preincubated in the low- or high-glucose environment before use. A total of 1.0×10^3^ cells per well were seeded in an ultralow attachment plate (3471, Corning) with a sphere formation medium composed of a serum-free DMEM/F12 (D8062, Sigma), 1% methylcellulose (M0512, Sigma), 20 ng/mL EGF (PHG0311, Thermo Fisher Scientific), 20 ng/mL FGF (PHG0266, Thermo Fisher Scientific), B-27 supplement (17504044, Thermo Fisher Scientific), and N-2 supplement (17502048, Thermo Fisher Scientific). Seven days after seeding, 4 visual fields were randomly chosen, and the sum of spheres with a diameter over 50 μm was calculated using a BZ-X700 microscope (Keyence, Osaka, Japan).

### Histological analysis and immunohistochemistry

Obtained specimens were fixed with paraformaldehyde and embedded in paraffin. For histopathological analysis, pancreatic tissue samples were sectioned at 3–5 μm and stained with hematoxylin and eosin (H&E). The whole slide was reconstructed as one image and analyzed using a BZ-X700 microscope (Keyence); the area and number of PanINs were calculated using a BZ-X Analyzer (Keyence).

For immunohistochemical analysis, sectioned pancreata were soaked in a target retrieval solution, pH 6 (2369, Dako, Jena, Germany) or pH 9 (2367, Dako), and steamed at 120°C for 5 min for antigen retrieval. A VECTASTAIN ABC kit (PK-4001, Vector Laboratories, CA, USA) was used according to the supplier’s protocol for a secondary antibody. The following antibodies were used: anti- phosphorylated STAT3 (pSTAT3) (rabbit monoclonal, 1:200, #9145, CST, MA, USA), anti-MYC (rabbit monoclonal, 1:100, ab32072, Abcam, Cambridge, UK), and anti-pERK (rabbit monoclonal, 1:400, #4370, CST).

The scoring system described below was used for immunostaining quantification: Total Score for Immunostaining = Quantitative Score + Intensity Score. Quantitative Score: 0%, no staining; 1, less than 20% staining; 2, 20–75% staining; and 3, more than 75% staining. Intensity Score: 0, no staining; 1, weak staining; 2, moderate staining; and 3, strong staining.

### Western blot analysis

Proteins were extracted using RIPA buffer containing protease and phosphatase inhibitors. Extracted proteins were separated by SDS-PAGE and transferred to nitrocellulose membranes. The blots were probed with the following antibodies: anti-MYC (rabbit monoclonal, 1:1,000, ab32072, Abcam), anti-pERK (rabbit monoclonal, 1:2,000, #4370, CST), anti-ERK (rabbit monoclonal, 1:1,000, #4695, CST), anti-beta-actin (mouse monoclonal, 1:10,000, A5316, Sigma), anti-pSTAT3 (rabbit monoclonal, 1:2,000, #9145, CST), anti-STAT3 (rabbit monoclonal, 1:2,000, #4904, CST), anti-Zeb1 (rabbit polyclonal, 1:500, NBT1-05987, Novus Biologicals, CO, USA), anti-pAKT (rabbit monoclonal, 1:2,000, #4060, CST), and anti-AKT (rabbit monoclonal, 1:1,000, #4691, CST).

### siRNAs

For the RNAi experiment, human siMYCs (s9130 and s9131, Thermo Fisher Scientific) and murine siMYCs (s70224 and s70225, Thermo Fisher Scientific) were used. The Lipofectamine RNA iMax transfection reagent (1378500, Thermo Fisher Scientific) was used for transfection. All procedures were carried out according to the manufacturer’s instructions.

### RT-PCR

Total RNA was extracted from pancreatic cancer cells and reverse transcribed to cDNA using a RNeasy kit (#74106, Qiagen, Hilden, Germany) and PrimeScript reverse transcriptase (#2680, Takara, Shiga, Japan). The following TaqMan assays were obtained from Applied Biosystems: human CDH1 (Hs01023895_m1), murine CDH1 (Mm01247357_m1), human CDH2 (Hs00983056_m1), murine CDH2 (Mm01162497_m1), human MYC (Hs00153408_m1), murine MYC (Mm00487804_m1), human Nanog (Hs02387400_g1), murine Nanog (Mm02019550_s1), human KLF4 (Hs00358836_m1), murine KLF4 (Mm00516104_m1), human SOX2 (Hs01053049_s1), murine SOX2 (Mm03053810_s1), human OCT4 (Hs04360467_gH), murine OCT4 (Mm03053917_g1), human beta-actin (Hs01060665_g1), and murine beta-actin (Mm02619580_g1). The Thunderbird probe qPCR mix (QPS-101, Toyobo, Osaka, Japan) was used for RT-PCR. Amplification of all samples was monitored using a QuantiStudio 6 Flex real-time PCR system (Thermo Fisher Scientific), and expression levels of target genes were normalized to those of beta-actin.

### Statistical analysis

All experiments were independently performed three or more times using biological replicates. Data are presented as the mean+s.d. Comparisons between biological replicates were performed using a two-tailed Student’s *t*-test, unless otherwise indicated in the figure legends. We set the significance level at P<0.05. For analyzing multiple groups, ANOVA was performed, and the factors that were considered significant were then independently tested with Tukey’s HSD test.

## Results

### STZ-induced hyperglycemia accelerates PanIN progression in mice

**Fig 1:**
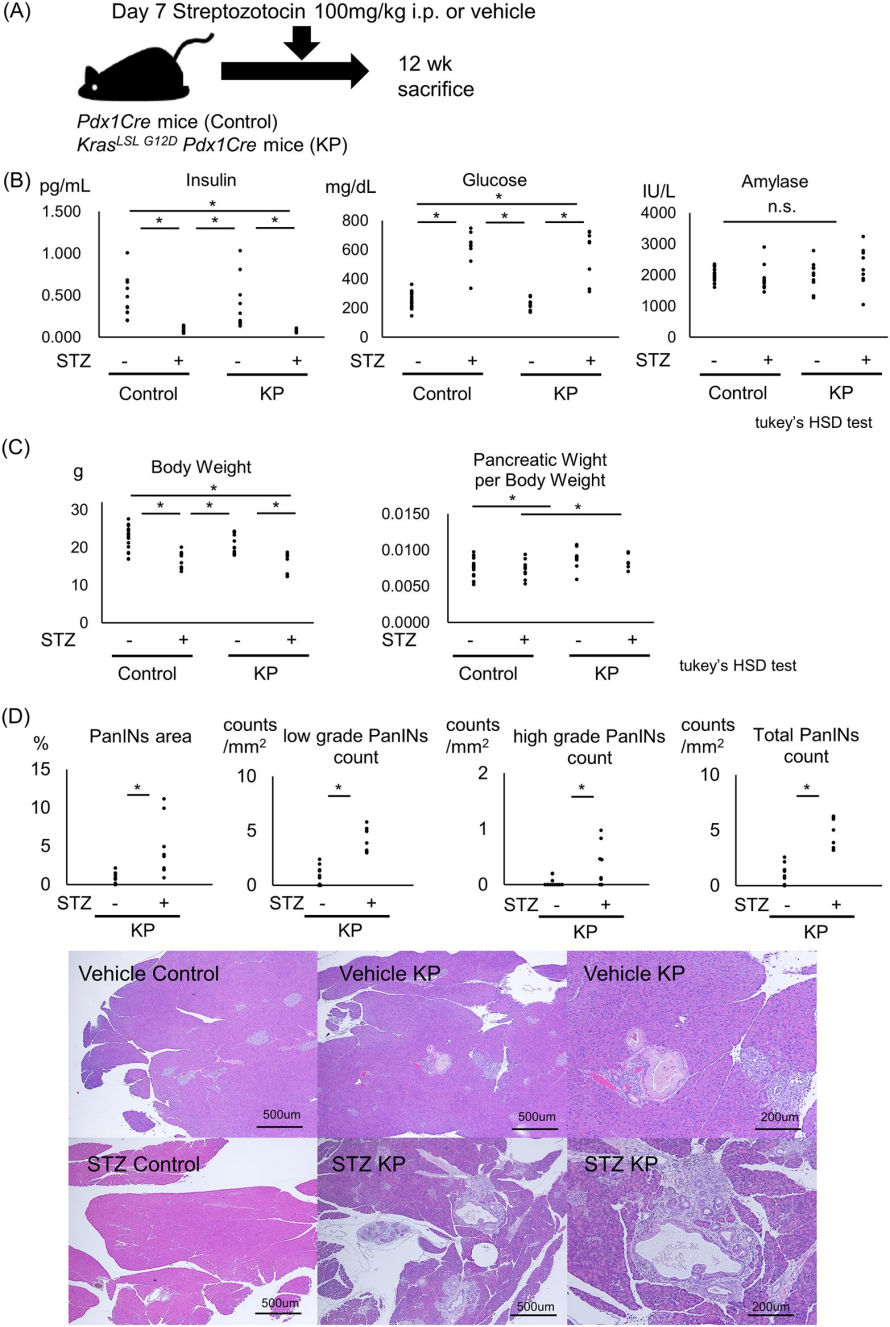
STZ-induced hyperglycemia promotes precancerous PanIN progression in *Kras*^*LSL G12D*^ *Pdx1Cre* mice. *Kras*^*LSL G12D*^ *Pdx1Cre* mice (KP) were injected with STZ 7 days after birth and sacrificed at 12 weeks of age (vehicle control: n = 18; vehicle KP: n = 10; STZ control: n = 11; and STZ KP: n = 8). (A) Scheme of the mouse model used and the associated injection schedule. (B) Blood levels of insulin, glucose, and amylase on the day of sacrifice (Tukey’s HSD test). (C) Body weight and the pancreatic weight-to-body weight ratio (Tukey’s HSD test). (D) Numbers of low-grade, high-grade, and total PanINs per 1 mm^2^ and percentages of the PanIN area. Representative hematoxylin and eosin (H&E)-stained sections from the vehicle control, STZ control, vehicle KP, and STZ KP mice. *P<0.05.

KP mice develop PanINs that gradually progress to pancreatic cancer and are known to mimic PanIN in humans [19]. To clarify the effects of hyperglycemia on PanINs and their progression, we intraperitoneally injected STZ dissolved in a citrate buffer or the citrate buffer alone (vehicle) into KP mice and *Pdx1Cre* mice (control) on postnatal day 7 to induce diabetes (Fig 1A). Four groups, the vehicle control, STZ control, vehicle KP, and STZ KP groups, were analyzed. STZ successfully induced type 1-like diabetes characterized by elevated blood glucose and decreased blood insulin levels. No elevation in the amylase level, a sign of pancreatitis, was detected in any of the four groups (Fig 1B). STZ injection reduced body weight in both control and KP mice at 12 weeks of age. Compared with the control mice in both the vehicle and STZ groups, KP mice had an increased ratio of pancreatic weight to body weight (Fig 1C). The percentage of the PanIN area at 12 weeks of age increased upon STZ-induced diabetes (control KP: 0.73 ± 0.69%, STZ KP: 4.8 ± 3.8%). The numbers of low-grade, high-grade, and total PanINs also increased in the STZ-induced KP mice (Fig 1D).

### Hyperglycemia enhances cell viability and sphere formation in Kras-mutant pancreatic ductal cells

**Fig 2:**
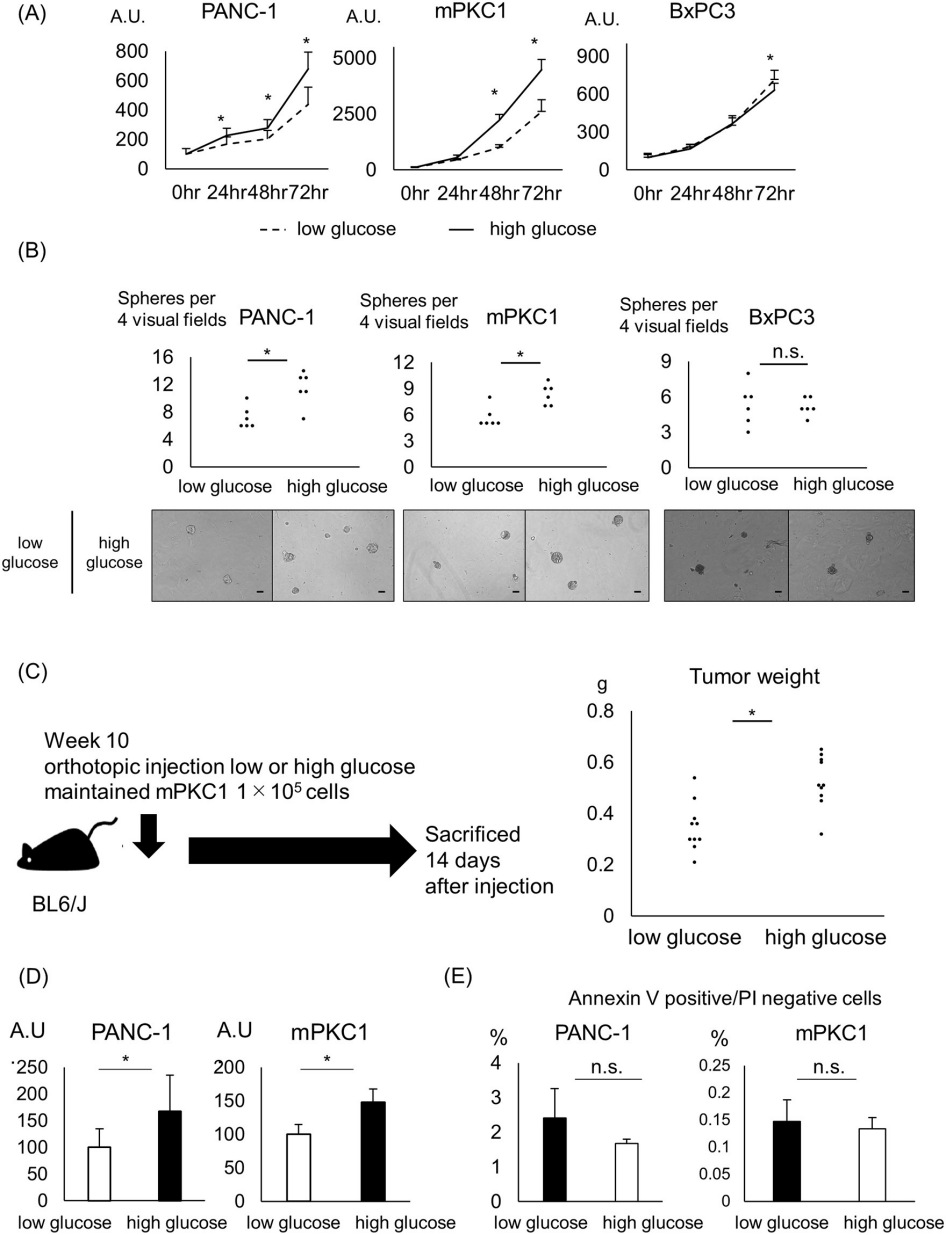
Increased cell viability and sphere formation in Kras-mutant pancreatic ductal cells in a hyperglycemic environment. The pancreatic ductal cancer cell lines PANC-1, mPKC1, and BxPC3 were maintained in a low-glucose (5.5 mM) or high-glucose (25 mM) DMEM for 28 days prior to the experiment. (A) Time course of the viability of pancreatic ductal cells grown in the low- or high-glucose medium, measured by the WST assay (n = 8 each). (B) Quantification of the sphere-forming capacity of pancreatic ductal cells 7 days after seeding (n = 6 each); Scale bar, 50 μm. (C) Murine-derived mPKC1 cells were orthotopically injected into the tail of the mouse pancreas. Tumor weight was measured 14 days after injection (n = 10 each). (D) BrDU cell proliferation assay of pancreatic ductal cells in the low- or high-glucose medium 48 hr after seeding (n = 8 each). (E) Annexin V assay of pancreatic ductal cells and the percentage of annexin V-positive/PI-negative cells (n = 3 each). Error bars: mean+s.d. *P<0.05.

To investigate the effect of hyperglycemia on pancreatic ductal cells in vitro, we used the pancreatic ductal cancer cell lines PANC-1, BxPC3, and mPKC1, a cell line generated from a primary pancreatic tumor of an *ElaCre Kras*^*LSL G12D*^ *trp53*^*fl/fl*^ *EYFP*^*Tg/Tg*^ mouse. PANC-1 and mPKC1 are cell lines with oncogenic Kras, and BxPC3 is a cell line with wild-type Kras. All cells were maintained in low- or high-glucose medium for 28 days prior to assessing the effect of hyperglycemia on the pancreatic ductal cells. In a WST assay, high glucose increased cell viability in Kras-mutant PANC-1 and mPKC1 cells (Fig 2A). In contrast, hyperglycemia did not increase cell viability in Kras-wild-type BxPC3 cells (Fig 2A). As in the WST assay, high glucose increased sphere formation 7 days after seeding only in Kras-mutant ductal cells (Fig 2B). Murine-derived mPKC1 cells were injected into the pancreatic tail of BL6/J mice to generate an orthotopic model. High-glucose medium-maintained mPKC1 cells formed larger tumors than their low-glucose medium-cultured counterparts (Fig 2C). The BrDU assay showed increased DNA synthesis in high-glucose medium-maintained cells, indicating that the high-glucose environment enhanced the proliferation compared with that in the low-glucose environment (Fig 2D). On the other hand, no change in the percentage of annexin V-positive and PI-negative cells was observed according to the glycemic change (Fig 2E). To examine whether the duration of incubation with a low or high glucose concentration affects cell viability and sphere formation, we performed the WST and sphere formation assays after 72 hr of incubation under the low- or high-glucose condition. In this short-term culture at the low or high glycemic level, the glucose concentration did not affect cell viability or sphere formation in pancreatic ductal cells (S2A and S2B Fig).

### High glucose enhances pSTAT3 and MYC expression in Kras-mutant pancreatic ductal cells and the STZ KP mouse model

**Fig 3:**
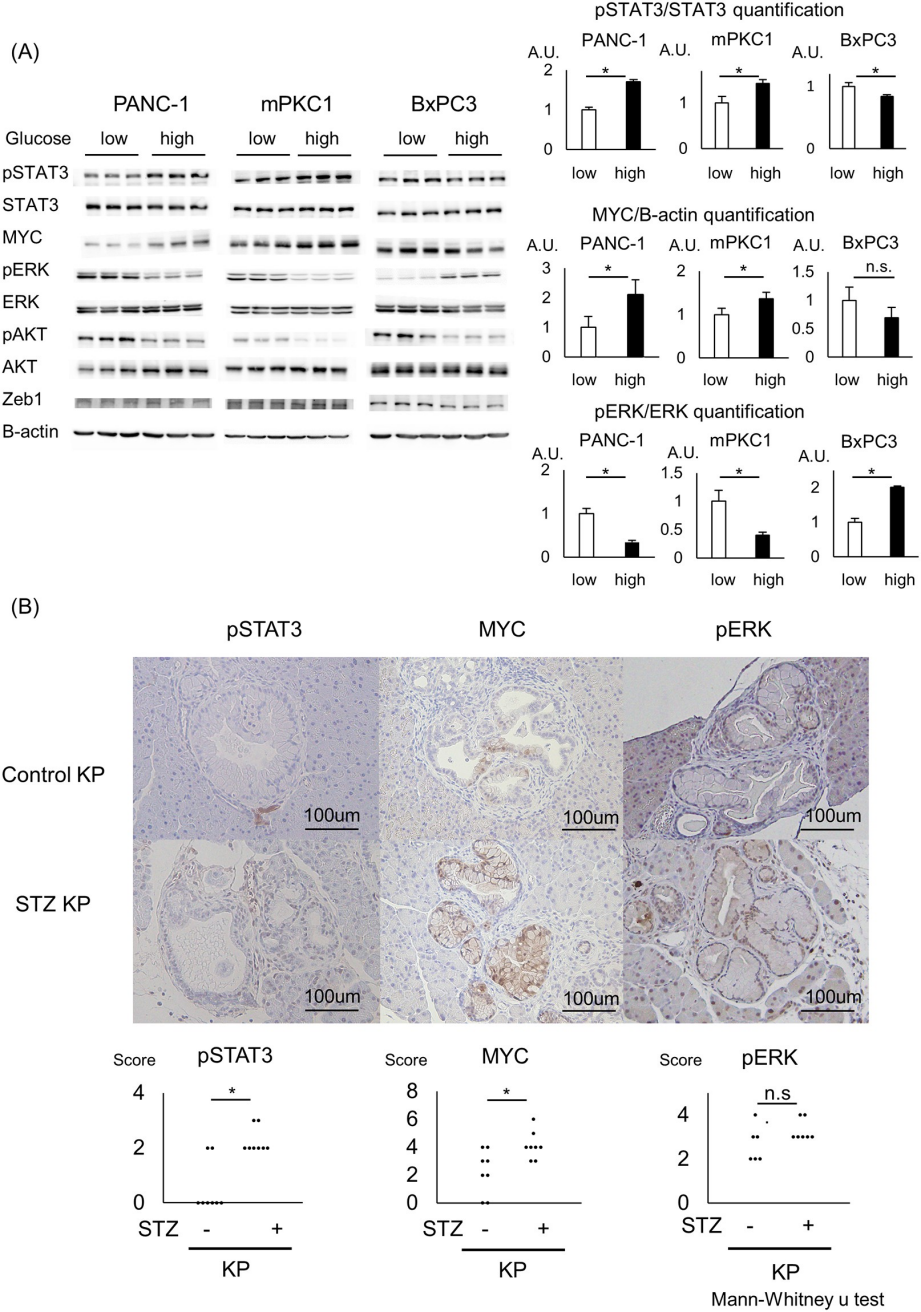
Hyperglycemia elevates MYC expression in combination with STAT3 activation. All pancreatic ductal cell lines (PANC-1, mPKC1, and BxPC3) were preconditioned with a low-glucose (5.5 mM) or high-glucose (25 mM) DMEM for 28 days. (A) Western blot analysis of the levels of pSTAT3, STAT3, MYC, pERK, ERK, pAKT, AKT, Zeb1, and beta-actin in the low- or high-glucose medium-maintained cells. Quantification of pSTAT3/STAT3, MYC/beta-actin, and pERK/ERK (n = 3 each); error bars: mean+s.d. (B) Immunostaining of control KP vs. STZ KP pancreata for pSTAT3, MYC, and pERK and the associated scoring. The pancreata from the STZ KP and vehicle KP mice were sectioned in advance. Scale bar, 200 μm; n = 8 each; *P<0.05.

To elucidate the underlying mechanism by which high-glucose conditions enhance cell viability and sphere formation, we examined proteins related to growth and tumorigenesis. For growth, a high-glucose environment produced increased phosphorylation of STAT3 but not ERK and AKT, the major effectors of the Kras pathway. For sphere formation-related proteins, the expression of Zeb1 did not differ according to the glucose concentration, while the expression of MYC was elevated with a high glucose level. These changes in protein expression levels were observed for PANC-1 and mPKC1, both of which are Kras-mutant cell lines, but not for BxPC3, a Kras-wild-type cell line (Fig 3A). The elevations in the pSTAT3 and MYC levels were only observed when cells were maintained in the high- or low-glucose medium for 28 days and not when cells underwent 72 hr of glycemic pretreatment (S2C Fig). We also examined the mRNA expression levels of EMT-related and pluripotency genes using RT-PCR. Although some gene expression levels changed after 4 weeks of the glycemic change, there were no common changes among the EMT-related and pluripotency genes (S3 Fig).

To clarify whether the changes in the expression of pSTAT3, MYC, and pERK were only in vitro phenomena or these changes play important roles in PanIN progression in vivo, we performed immunostaining for pSTAT3, MYC, and pERK in precancerous PanINs, with or without hyperglycemia. The levels of pSTAT3 and MYC were higher in the STZ KP PanINs than in the control KP PanINs; however, staining for pERK did not change depending on the glycemic background (Fig 3B).

### STAT3 inhibition in Kras-mutant pancreatic ductal cells blocks the cellular changes induced by a high-glucose environment

**Fig 4:**
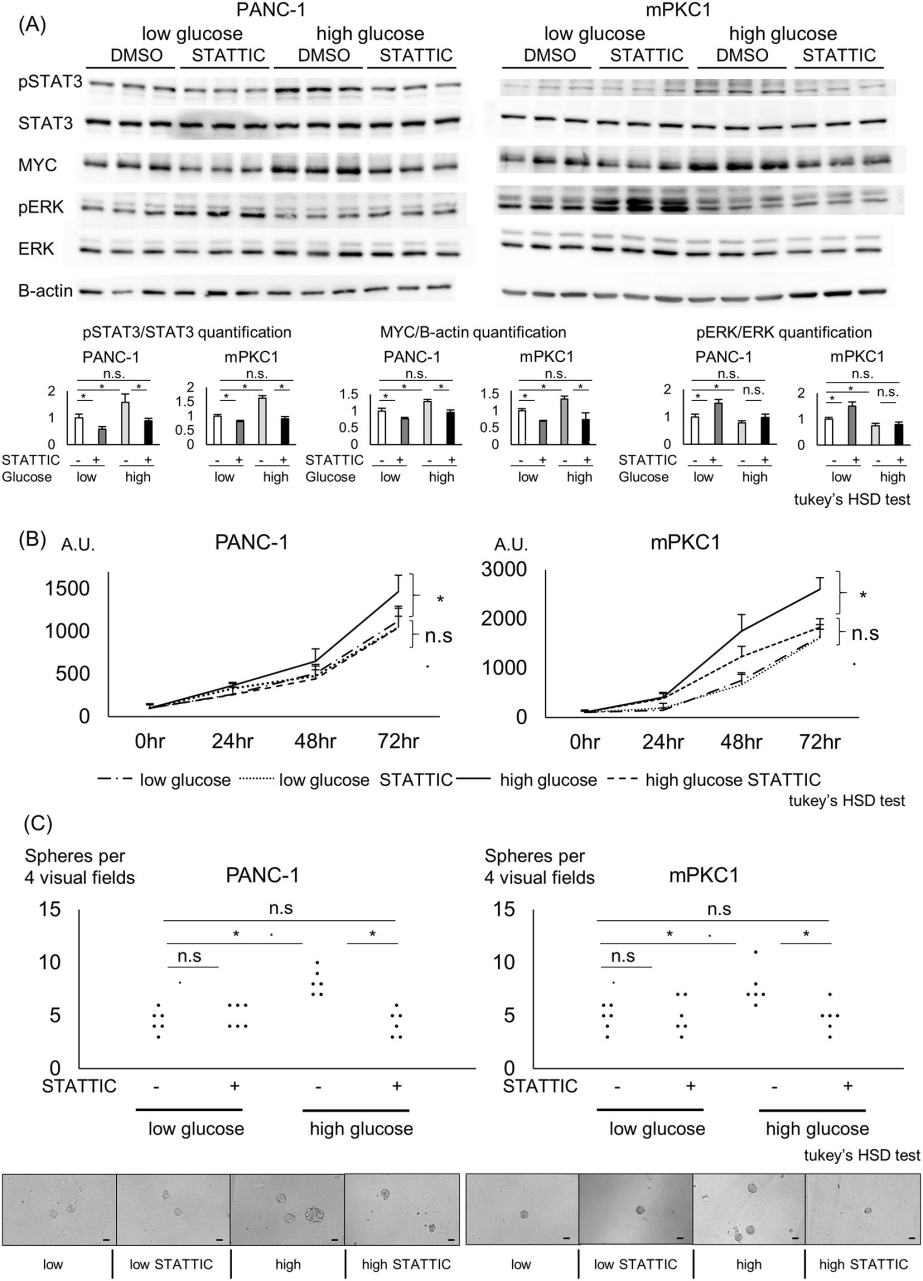
STAT3 inhibition and its relationship with MYC in pancreatic ductal cells. Kras-mutant PANC-1 and mPKC1 cells were incubated with a low-glucose (5.5 mM) or high-glucose (25 mM) DMEM for 28 days. The cells were then treated with 1 μM STAT3 inhibitor STATTIC. (A) Western blot analysis of PANC-1 and mPKC1 cells with or without 1 μM STATTIC treatment. The levels of pSTAT3, STAT3, MYC, pERK, ERK, and beta-actin are shown. Quantification of pSTAT3/STAT3, MYC/beta-actin, and pERK/ERK (n = 3 each); error bars: mean+s.d. (B) Time course of the viability of PANC-1 and mPKC1 cells incubated with or without 1 μM STATTIC, measured by the WST assay (n = 8 each); error bars: mean+s.d. (C) Quantification of the sphere-forming capacity of PANC-1 and mPKC1 cells with or without 1 μM STATTIC treatment (n = 6 each); Scale bar, 50 μm; Tukey’s HSD test; *P<0.05.

We used STATTIC, a selective STAT3 inhibitor, to uncover the relation between STAT3 and MYC in Kras-mutant pancreatic ductal cell lines. STATTIC at 1 μM successfully inhibited STAT3 phosphorylation in the Kras-mutant pancreatic ductal cell lines, blocking the increase induced by high-glucose conditions (Fig 4A). The elevation in MYC expression was also reversed upon STAT3 inhibition, showing the potential of STAT3 as an upstream regulator of MYC. The decreases in both pSTAT3 and MYC expression were more distinct in high-glucose medium-maintained cells than in low-glucose medium-maintained cells. In low-glucose-maintained cells, the phosphorylation of ERK was elevated upon STATTIC treatment, showing an inverse relation with the phosphorylation of STAT3 (Fig 4A). To show whether enhanced cell viability and sphere formation under a high-glucose environment are also reversed by STAT3 inhibition, we employed STATTIC to treat low- or high-glucose medium-maintained cells and performed WST and sphere formation assays. STATTIC attenuated cell viability and sphere formation in the high-glucose medium-maintained Kras-mutant cells to levels comparable to those seen in cells in a low-glucose environment. STATTIC did not affect cell viability or sphere formation in low-glucose medium-maintained cells (Fig 4B and 4C).

### MYC knockdown inhibited sphere formation in Kras-mutant pancreatic ductal cells

**Fig 5:**
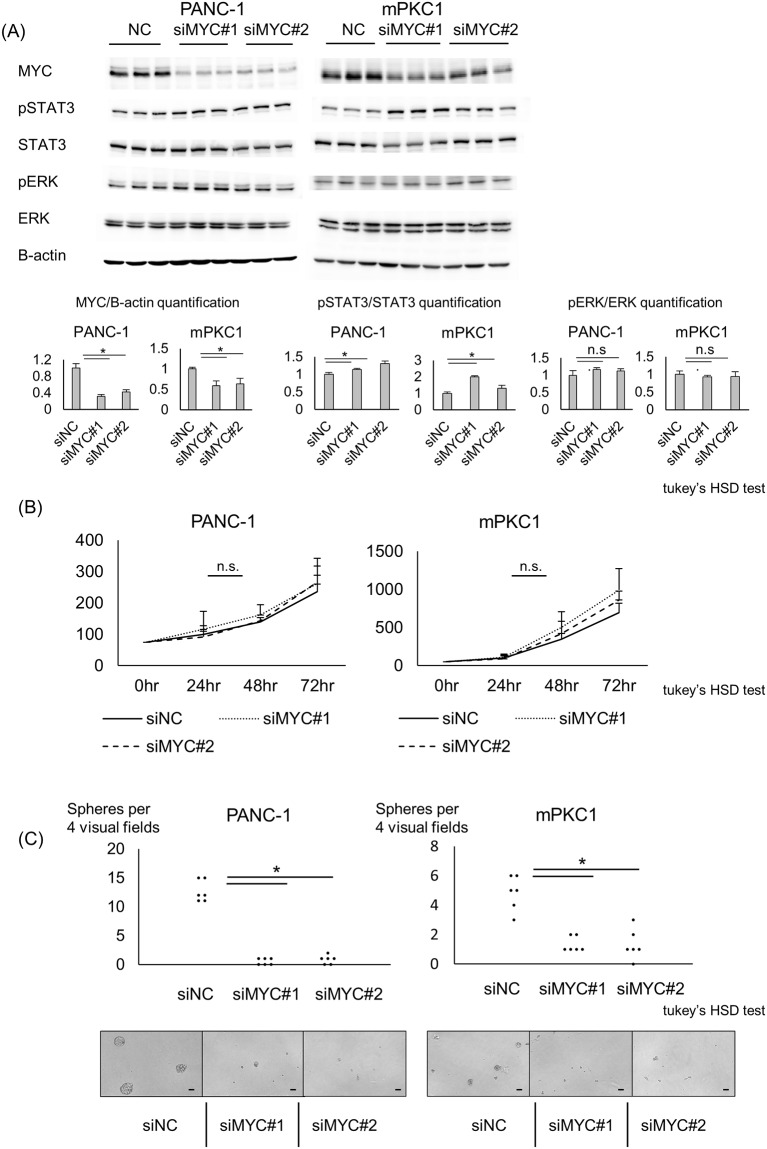
Knockdown of MYC expression decreases sphere formation in pancreatic ductal cells. Kras-mutant PANC-1 and mPKC1 cells were maintained in the high-glucose medium, and siMYC was used to knock down the MYC expression. (A) Western blot analysis of ductal cells after siMYC transfection. The levels of pSTAT3, STAT3, MYC, pERK, ERK, and beta-actin are shown. Quantification of MYC/beta-actin, pSTAT3/STAT3, and pERK/ERK (n = 3 each); error bars: mean+s.d. (B) WST assay of MYC-knockdown PANC-1 and mPKC1 cells at 24, 48, and 72 hr after the transfection (n = 8 each); error bars: mean+s.d. (C) Quantification of the sphere-forming capacity of pancreatic ductal cell lines after knockdown of MYC expression (n = 6 each); Scale bar, 50 μm; Tukey’s HSD test; *P<0.05.

We knocked down the MYC expression using siMYC in high-glucose medium-maintained PANC-1 and mPKC1 cells. In both ductal cell lines, MYC expression was successfully knocked down (Fig 5A). Upon knockdown of MYC expression, STAT3 phosphorylation was slightly elevated, but no change was observed in the ERK phosphorylation (Fig 5A), indicating that STAT3 may receive a negative feedback from MYC activation. We then evaluated the effects of MYC on cell growth and sphere formation. The decreased expression of MYC did not affect the cell viability within 72 hr (Fig 5B). Sphere formation 7 days after seeding was dramatically decreased after knocking down MYC expression (Fig 5C).

## Discussion

In the present study, hyperglycemia accelerated PanIN progression, with increases in pSTAT3 and MYC levels. STAT3 is known to be an upstream regulator of MYC [20, 21], and its association with hyperglycemia has been reported in the kidneys [22], lungs [23], and heart [24]. In renal mesangial cells, hyperglycemia was shown to enhance the activation of STAT3 [25]. STAT3 activation has also been reported to promote PanIN formation in an oncogenic Kras mouse model, showing the importance of STAT3 in the development of pancreatic cancer [26]. MYC reprograms pancreatic cancer metabolism, independent of MAPK signaling and the MAPK effector ERK [27]. Oncogenic Ras stabilizes MYC by phosphorylation of serine-62, thereby supporting MYC activation [28]. In mice with oncogenic Kras, a heterogenic knockout of MYC reduces the development of pancreatic cancer, and physiological elevation of MYC, induced by the insertion of two copies of ROSA-driven MYC, significantly worsened the survival in a mouse model of pancreatic cancer [29, 30]. In other words, relatively small alterations in the expression level of MYC can work synergistically with oncogenic Kras to promote PanIN progression, independent of ERK.

Using our hyperglycemic model, we showed that STAT3 might be an upstream regulator of MYC expression and might control both cell viability and sphere formation in Kras-mutant cell lines. In MYC knockdown experiments, we showed that the sphere formation capacity under STAT3 strongly relies on MYC expression. However, siMYC did not decrease cell viability. Phosphorylation of STAT3 was slightly elevated upon MYC knockdown (Fig 5A), and this elevation may be canceling the MYC knockdown-induced decrease in cell viability. Together, STAT3 is an important upstream regulator of MYC, but further studies are needed to elucidate whether it controls STAT3-promoted cell viability. Considering that the STAT3 inhibitor STATTIC decreased cell viability and sphere formation only in high-glucose medium-maintained cells with enhanced pSTAT3 expression, high-glucose medium-cultured cells are suspected to be more dependent on STAT3 and MYC than low-glucose medium-maintained cells in regard to cell growth and tumorigenesis. From this point of view, STAT3 and its effector MYC may be a pancreatic cancer driver in hyperglycemia. We did not treat the STZ-induced KP mice with STATTIC. However, taking into account a previous report, showing that STAT3 inhibition attenuated PanIN formation in KP mice [26], we suspect that STAT3 inhibition has the potential to attenuate PanIN progression, which is induced by STZ-induced hyperglycemia.

STAT3 and ERK showed an inverse relation under hyperglycemic conditions and STAT3 inhibition. An inverse relation between pERK and pSTAT3 in Kras-mutant pancreatic cancer cells has been reported recently, supporting our data [31]. Upon treatment with the STAT3 inhibitor STATTIC, low-glucose-maintained cells showed a stronger tendency toward enhanced ERK phosphorylation than did high-glucose-maintained cells. The proliferation of low-glucose-maintained cells is suppressed compared with that of high-glucose-maintained cells. When STAT3, a major growth signal, is suppressed in cells growing under low glycemic conditions, we suspect that more stress is induced compared with that in cells whose growth is enhanced under high-glucose conditions, resulting in stronger ERK phosphorylation.

Under low-glucose preconditioning, STAT3 phosphorylation was weakened in Kras-mutant cells (Fig 3A). However, despite the suppressed cell viability, phosphorylation of ERK and AKT was enhanced in low-glucose-maintained Kras-mutated cells compared with that in high-glucose-maintained cells (Fig 3A). Upon inhibition of AKT with MK2206 2HCL, the viability of low-glucose-maintained cells further dramatically decreased, showing that AKT acts as a growth signal even in these cell lines (S4A and S1B Figs). AKT inhibition also enhanced ERK phosphorylation, similar to that observed with the STAT3 inhibitor STATTIC. Based on these data, it is speculated that the phosphorylation of AKT and ERK is increased, in a compensatory manner, in low-glucose-maintained Kras-mutated cells.

Several reports have shown the effect of a hyperglycemic environment on pancreatic cancer. A high-glucose environment without glycemic pretreatment triggers an invasive character with an elevation in pERK expression in pancreatic cancer cells [32, 33]. When incubated for more than 4 weeks in high- or low-glucose medium, high-glucose medium-pretreated Kras-mutant pancreatic cancer cells show an enhanced metastatic ability, while low-glucose medium-maintained cells show a stronger tendency toward local invasion [34]. In our study, 72-hr incubation in a low- or high-glucose medium produced no changes in cell viability or sphere formation; however, after 28 days of pretreatment, the cell characteristics dramatically changed, with the promotion of cell proliferation and tumorigenesis. A prolonged duration of hyperglycemia is a reported risk factor for pancreatic cancer, and we showed here that exposure to a high-glucose environment for 28 days induced enhanced cell viability and sphere formation. Conclusively, long-term changes in glycemic levels may select cells adjusted to hyperglycemia with a relatively strong tendency toward uncontrollable growth, a widely known characteristic of cancers.

Pancreatic cancer and metabolic disorders, including diabetes mellitus, are closely related. In clinical patients, hyperglycemia often coexists with obesity, dyslipidemia, and other metabolic disorders. We focused on hyperglycemia as one of these metabolic risk factors of pancreatic cancer and demonstrated that hyperglycemia is an enhancer of PanIN progression. We showed in our oncogenic Kras mouse models that STZ-induced diabetes, which is characterized by high glucose and low insulin levels, aggravated PanIN progression. Streptozotocin is a cytotoxic agent and may itself be tumorigenic. However, STZ is reported to affect only cells with the glut2 transporter, which is not expressed in ducts and acinar cells of the pancreas. On postnatal day 7, an age when we used STZ in mice, our KP mouse models were expected to have no PanINs. Therefore, we think that the cellular toxicity to PanINs must be limited. Good control of hyperglycemia may contribute to an improved prognosis, preventing the progression of PanIN into pancreatic cancer. Little is known about the intimate relations between metabolic disorders and pancreatic cancer. Further understanding of these relations may lead to an earlier diagnosis and improved prognosis of this deadly cancer.

## Supporting information

S1 Fig Establishment of the murine pancreatic cancer cell line mPKC1. mPKC1 was established from a pancreatic tumor developed in an *ElaCre Kras*^*LSL G12D*^ *trp53*^*fl/fl*^ *EYFP*^*Tg/Tg*^ mouse. (A) Pancreas-specific expression of EYFP was detected in mPKC1 cells using a fluorescence microscope BZ-X700 (Keyence). (B) Orthotopic injection of mPKC1 to the pancreatic tail of BL6/J mice and tumor detection with micro-CT and H&E staining.
(TIF)

S2 Fig Hyperglycemia without pretreatment does not affect cellular characteristics of Kras-mutant pancreatic ductal cells. PANC-1, mPKC1, and BxPC3 cells were maintained under low- or high-glucose conditions for 72 hr prior to analysis. (A) Time course of the viability of pancreatic ductal cells in a low- or high-glucose medium, measured by the WST assay (n = 8 each). Error bars: mean+s.d. (B) Quantification of the sphere-forming capacity of pancreatic ductal cells 7 days after seeding (n = 6 each); Scale bar, 50 μm. (C) Western blot analysis of ductal cells. The levels of pSTAT3, STAT3, MYC, pERK, ERK, and beta-actin are shown. *P<0.05.
(TIF)

S3 Fig RT-PCR analysis of pancreatic ductal cells after 72 hr or 28 days of glycemic preconditioning. PANC-1, mPKC1, and BxPC3 cells were maintained under low- or high-glucose conditions for 72 hr or 28 days prior to analysis. The expression of CDH1, CDH2, Nanog, MYC, SOX2, KLF4, OCT4, and beta-actin was analyzed. The relative expression, normalized to that of beta-actin, is shown in arbitrary units (n = 3 each); error bars: mean+s.d. *P<0.05.
(TIF)

S4 Fig AKT inhibition and its effect on low glucose-maintained pancreatic ductal cells. Kras-mutant PANC-1 and mPKC1 cells were incubated with a low-glucose (5.5 mM) DMEM for 28 days. The cells were treated with 10 μM AKT inhibitor MK2206 2HCL. (A) Western blot analysis of PANC-1 and mPKC1 cells with or without 10 μM MK2206 2HCL treatment. The levels of pSTAT3, STAT3, pAKT, AKT, pERK, ERK, and beta-actin are shown. (B) Time courses of PANC-1 and mPKC1 cells incubated with or without 10 μM MK2206 2HCL, as measured by the WST assay (n = 8 each); error bars: mean+s.d. *P<0.05.
(TIF)

S1 Raw images (PDF)
