# Supplementary figures and images for: Hyperglycemia enhances pancreatic cancer progression accompanied by elevations in phosphorylated STAT3 and MYC levels
Source: PLoS One. 2020 Jul 1;15(7):e0235573. doi: 10.1371/journal.pone.0235573 (PMC7329089; doi:10.1371/journal.pone.0235573)

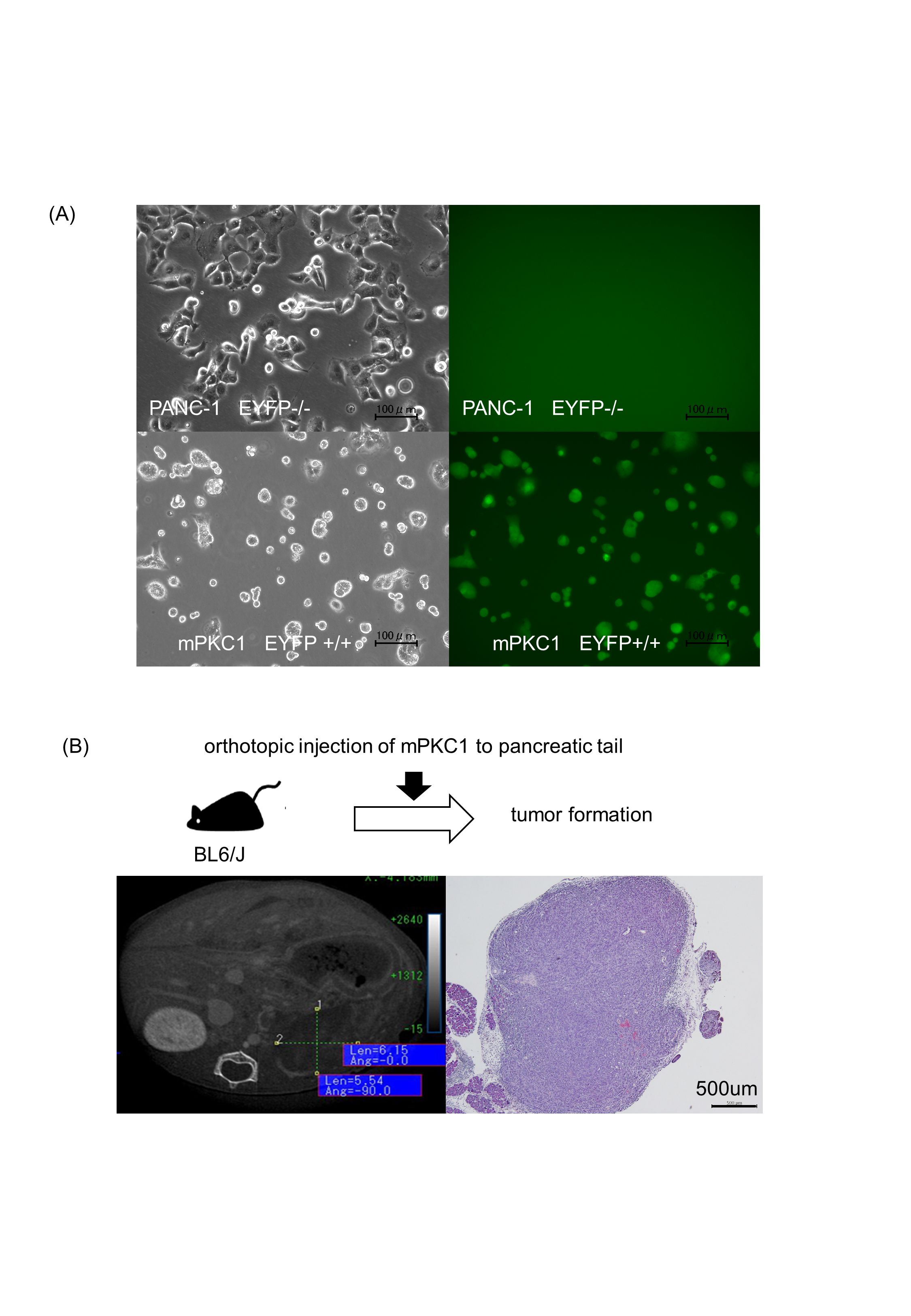

Supplement: S1 Fig — mPKC1 was established from a pancreatic tumor developed in an ElaCre KrasLSL G12D trp53fl/fl EYFPTg/Tg mouse. (A) Pancreas-specific expression of EYFP was detected in mPKC1 cells using a fluorescence microscope BZ-X700 (Keyence). (B) Orthotopic injection of mPKC1 to the pancreatic tail of BL6/J mice and tumor detection with micro-CT and H&E staining. (TIF) [file pone.0235573.s001.TIF]

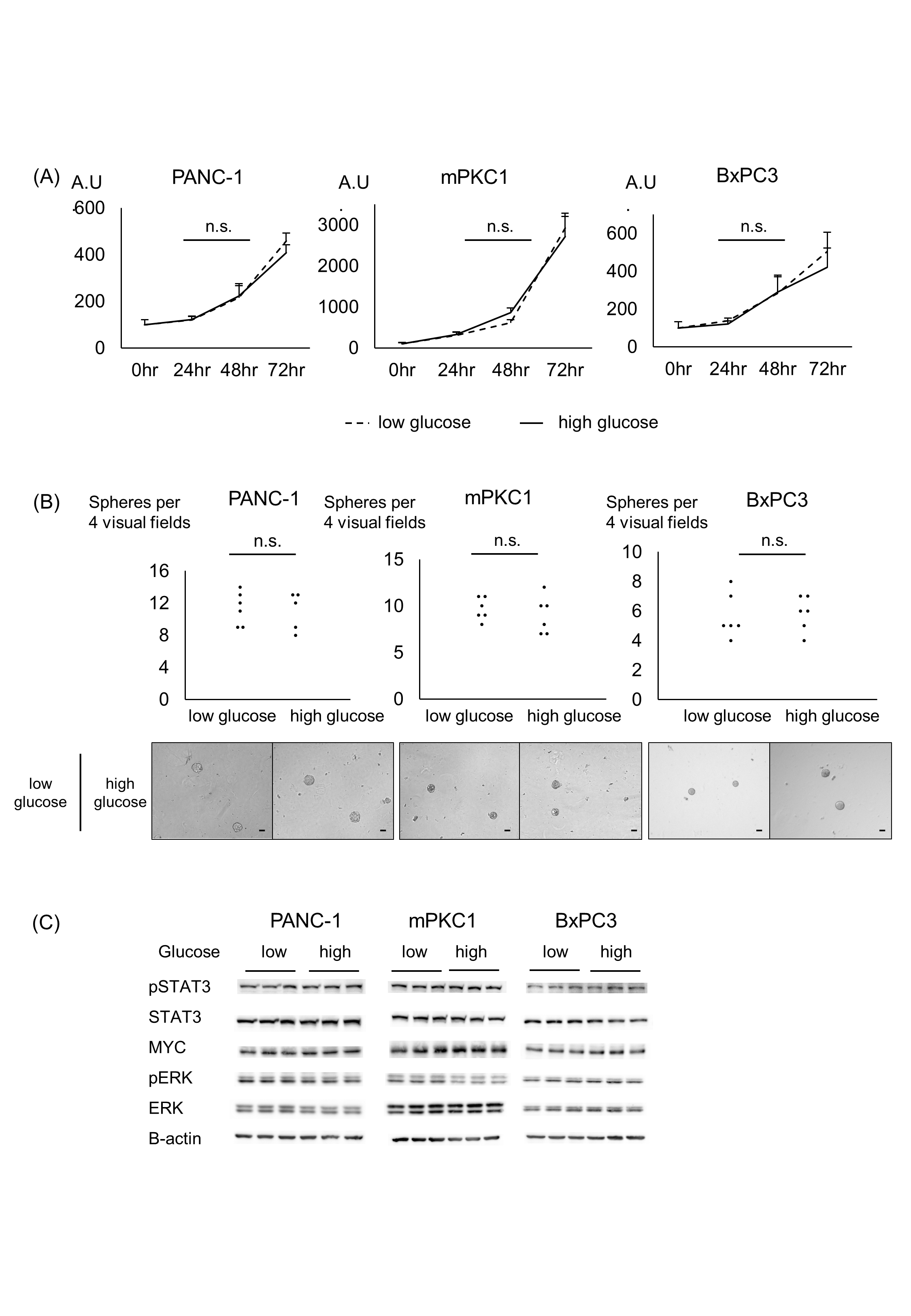

Supplement: S2 Fig — PANC-1, mPKC1, and BxPC3 cells were maintained under low- or high-glucose conditions for 72 hr prior to analysis. (A) Time course of the viability of pancreatic ductal cells in a low- or high-glucose medium, measured by the WST assay (n = 8 each). Error bars: mean+s.d. (B) Quantification of the sphere-forming capacity of pancreatic ductal cells 7 days after seeding (n = 6 each); Scale bar, 50 μm. (C) Western blot analysis of ductal cells. The levels of pSTAT3, STAT3, MYC, pERK, ERK, and beta-actin are shown. *P<0.05. (TIF) [file pone.0235573.s002.TIF]

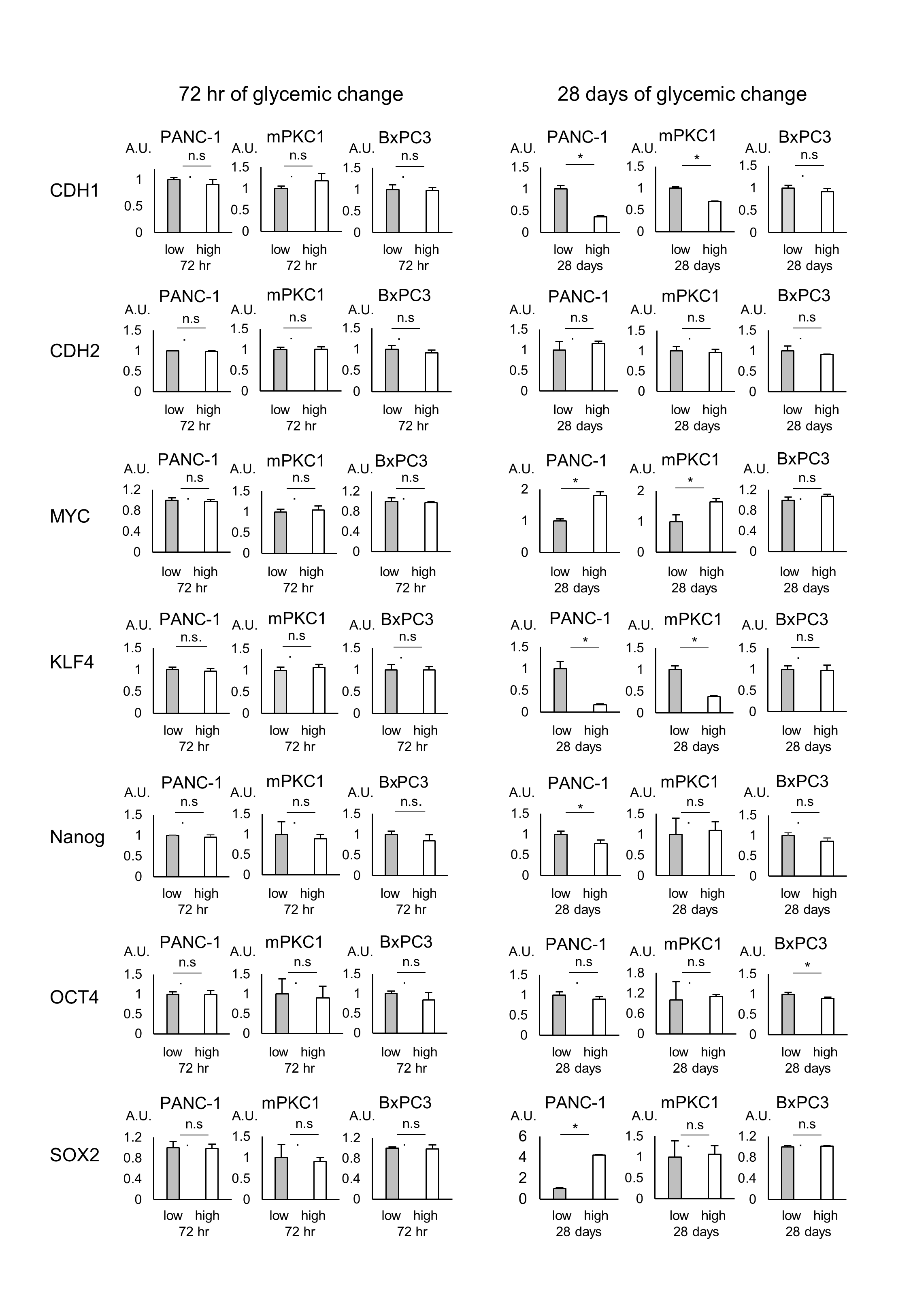

Supplement: S3 Fig — PANC-1, mPKC1, and BxPC3 cells were maintained under low- or high-glucose conditions for 72 hr or 28 days prior to analysis. The expression of CDH1, CDH2, Nanog, MYC, SOX2, KLF4, OCT4, and beta-actin was analyzed. The relative expression, normalized to that of beta-actin, is shown in arbitrary units (n = 3 each); error bars: mean+s.d. *P<0.05. (TIF) [file pone.0235573.s003.TIF]

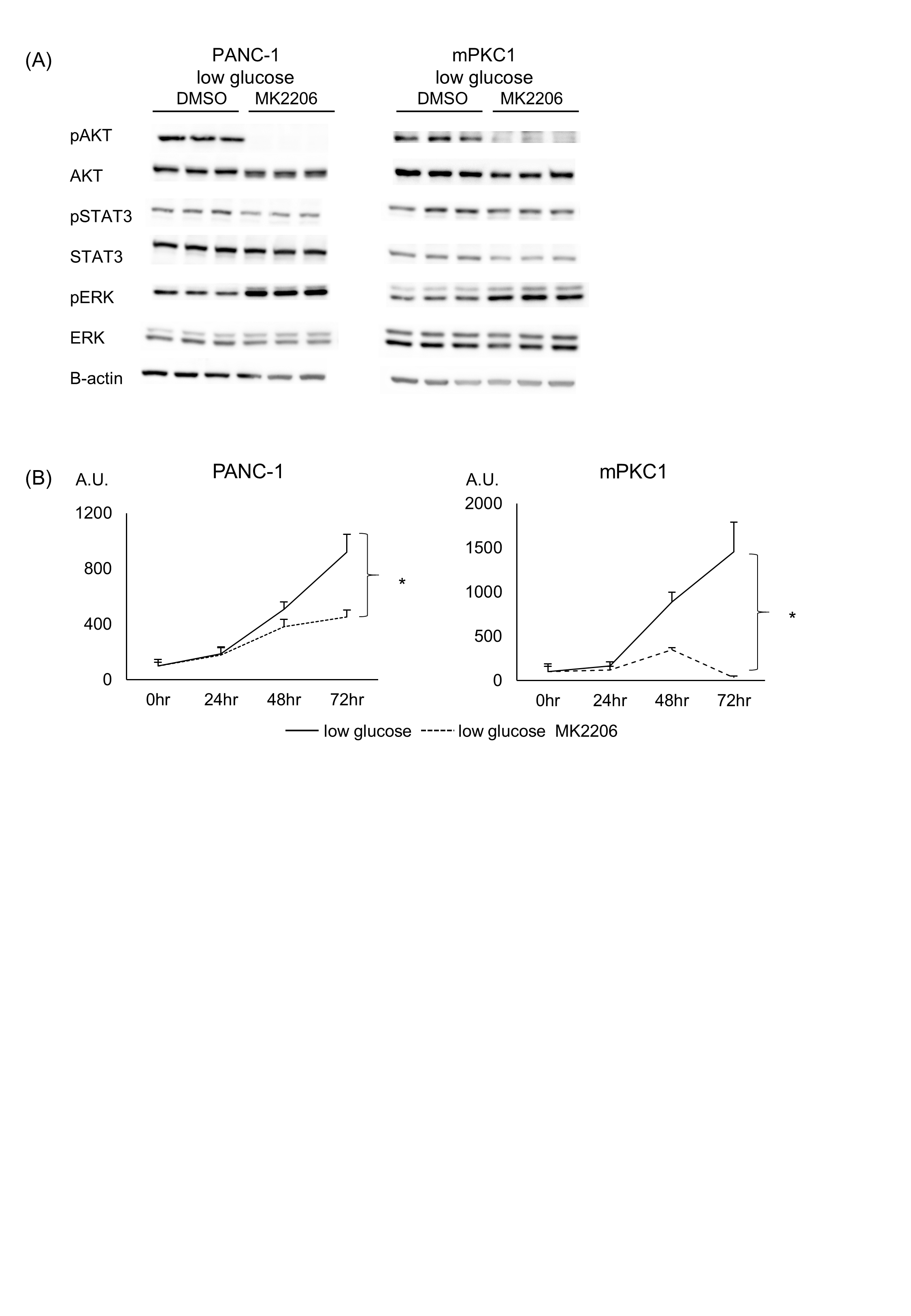

Supplement: S4 Fig — Kras-mutant PANC-1 and mPKC1 cells were incubated with a low-glucose (5.5 mM) DMEM for 28 days. The cells were treated with 10 μM AKT inhibitor MK2206 2HCL. (A) Western blot analysis of PANC-1 and mPKC1 cells with or without 10 μM MK2206 2HCL treatment. The levels of pSTAT3, STAT3, pAKT, AKT, pERK, ERK, and beta-actin are shown. (B) Time courses of PANC-1 and mPKC1 cells incubated with or without 10 μM MK2206 2HCL, as measured by the WST assay (n = 8 each); error bars: mean+s.d. *P<0.05. (TIF) [file pone.0235573.s004.TIF]
