# Supplementary material for: Hyperglycemia enhances pancreatic cancer progression accompanied by elevations in phosphorylated STAT3 and MYC levels
Source: PLoS One. 2020 Jul 1;15(7):e0235573. doi: 10.1371/journal.pone.0235573 (PMC7329089; doi:10.1371/journal.pone.0235573)

Raw Data of Gel blots

Raw Data of figures below are shown in order. All blots were captured with high sensitivity mode of FUSION Solo S (Vilber Lourmat, Collégien, France) .

Fig 3A PANC-1, Fig 3A mPKC1, Fig 3A BxPC3

Fig 4A PANC-1, Fig 4B mPKC1

Fig 5A PANC-1, Fig 5A mPKC1

S2. Fig C PANC-1 mPKC1, S2. Fig C BxPC3

S4. Fig A PANC-1, S4. Fig A mPKC1

Fig 3A PANC-1

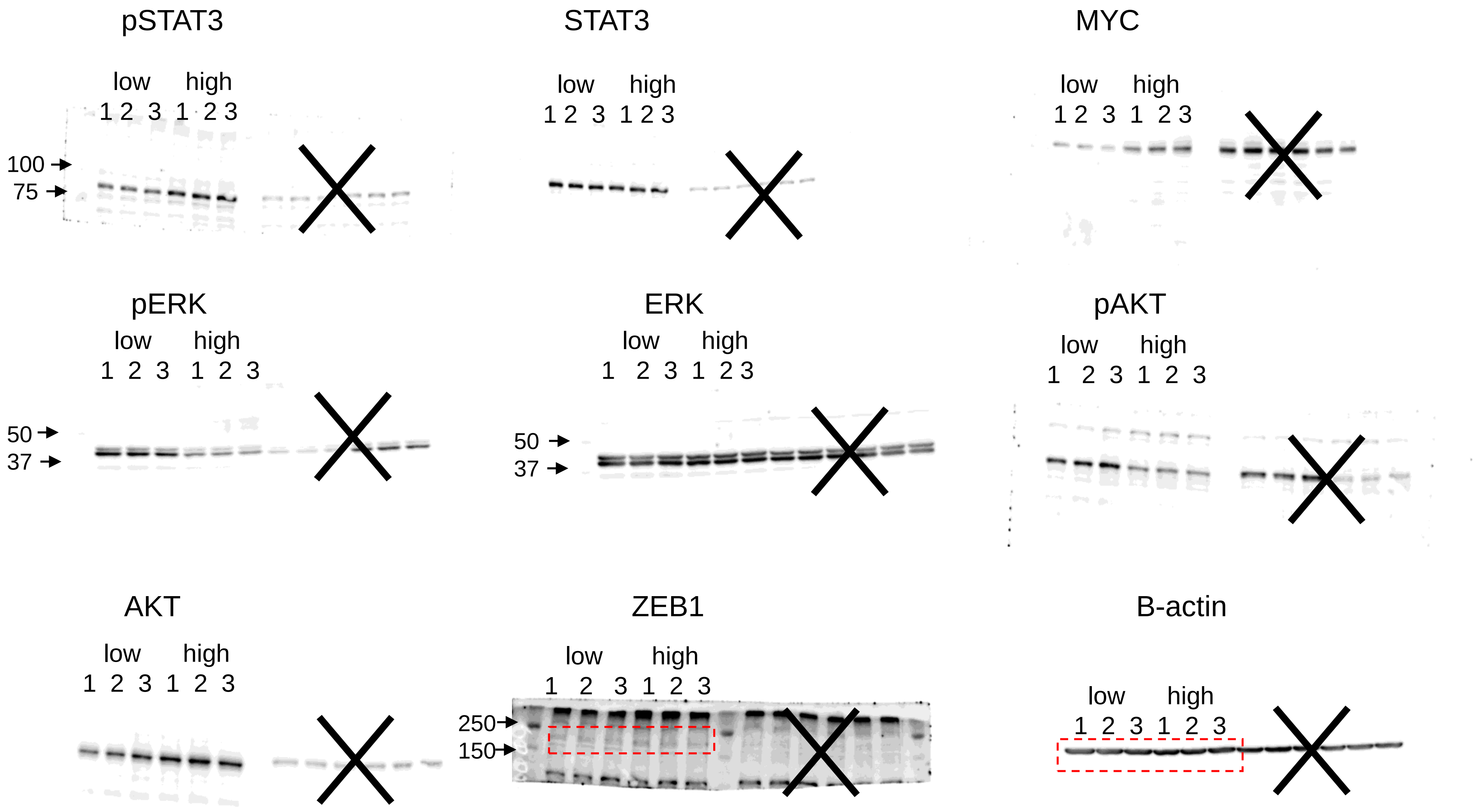
pSTAT3
low high
1 2 3 1 2 3
100
75
STAT3
low high
1 2 3 1 2 3
MYC
low high
1 2 3 1 2 3
pERK
low high
1 2 3 1 2 3
50
37
ERK
low high
1 2 3 1 2 3
50
37
pAKT
low high
1 2 3 1 2 3
AKT
low high
1 2 3 1 2 3
ZEB1
low high
1 2 3 1 2 3
250
150
B-actin
low high
1 2 3 1 2 3

Fig 3A mPKC1

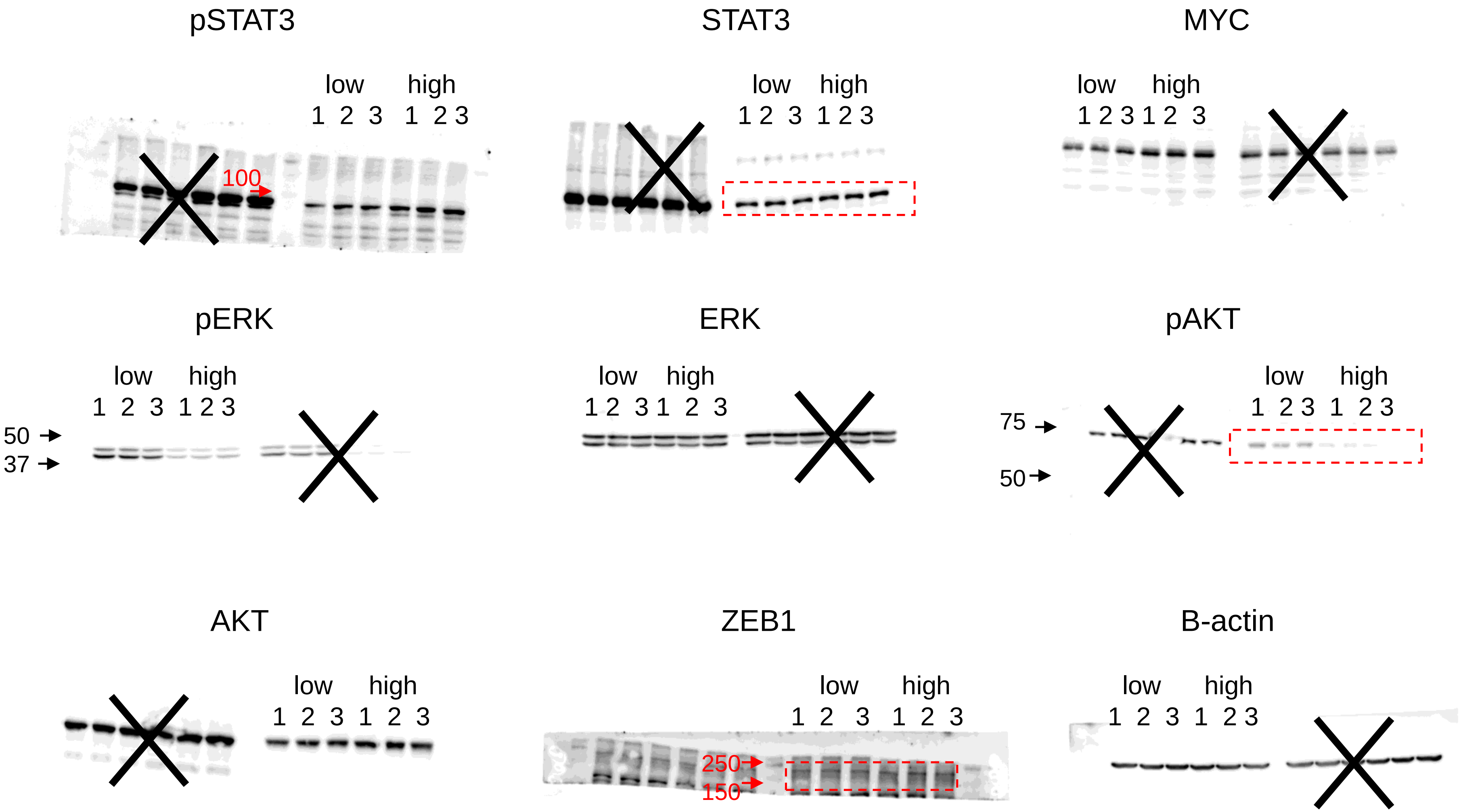
pSTAT3
low high
1 2 3 1 2 3
100
STAT3
low high
1 2 3 1 2 3
MYC
low high
1 2 3 1 2 3
pERK
low high
1 2 3 1 2 3
50
37
ERK
low high
1 2 3 1 2 3
pAKT
low high
1 2 3 1 2 3
75
50
AKT
low high
1 2 3 1 2 3
ZEB1
low high
1 2 3 1 2 3
250
150
B-actin
low high
1 2 3 1 2 3

Fig 3A BxPC3

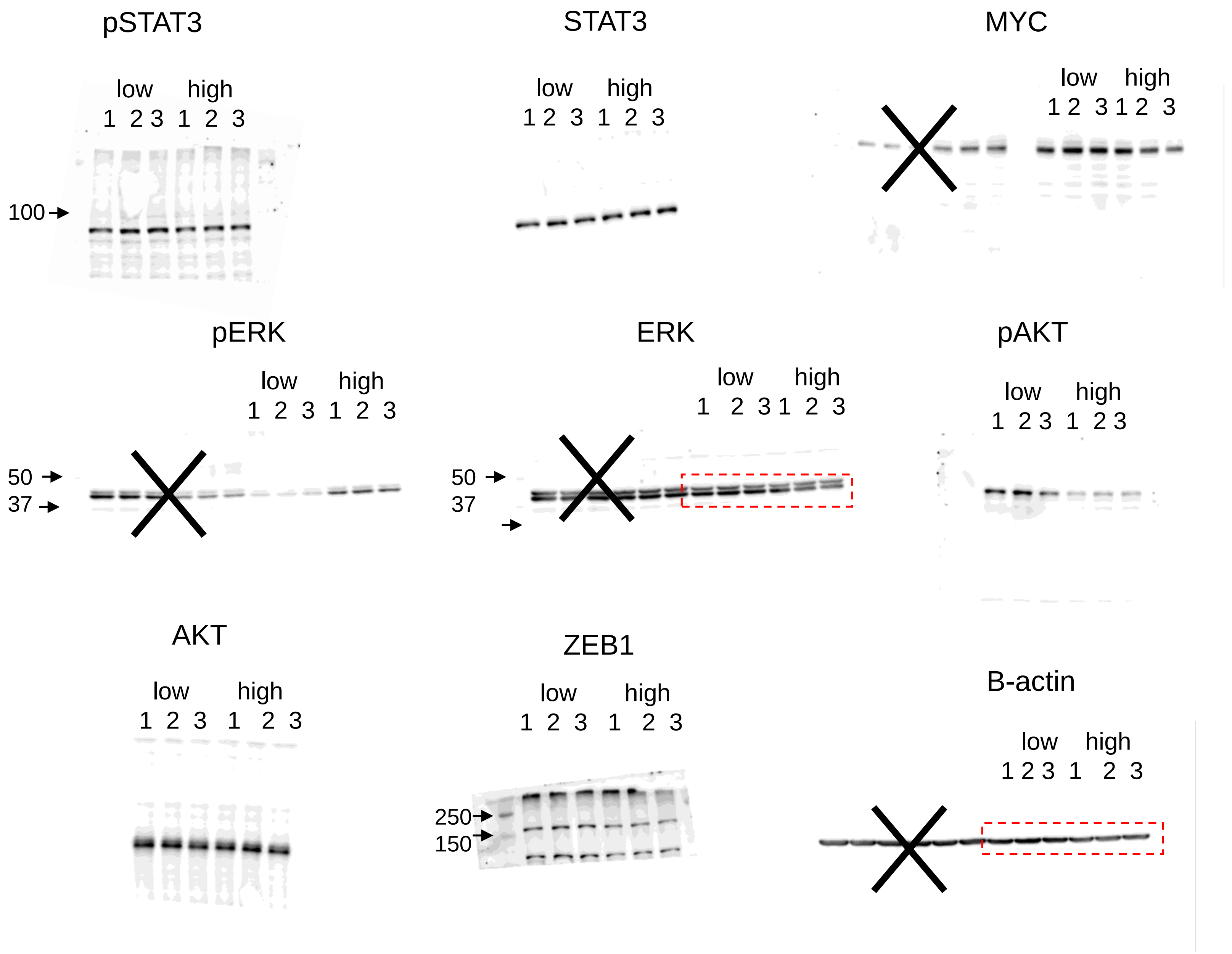
pSTAT3
low high
1 2 3 1 2 3
100
STAT3
low high
1 2 3 1 2 3
MYC
low high
1 2 3 1 2 3
pERK
low high
1 2 3 1 2 3
50
37
ERK
low high
1 2 3 1 2 3
50
37
pAKT
low high
1 2 3 1 2 3
AKT
low high
1 2 3 1 2 3
ZEB1
low high
1 2 3 1 2 3
250
150
B-actin
low high
1 2 3 1 2 3

Fig 4A PANC-1

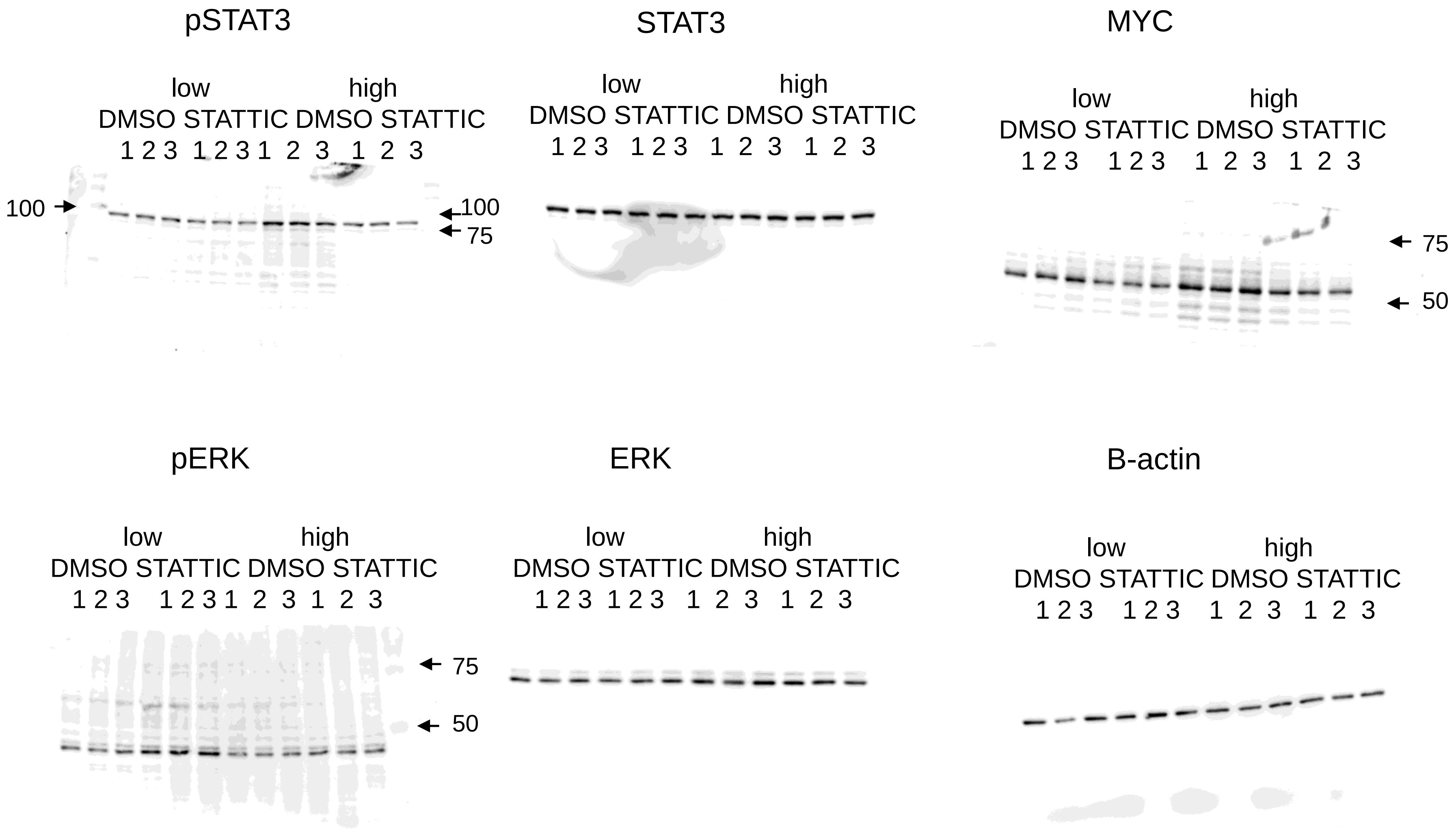
pSTAT3
low high
DMSO STATTIC DMSO STATTIC
1 2 3 1 2 3 1 2 3 1 2 3
100
100
75
STAT3
low high
DMSO STATTIC DMSO STATTIC
1 2 3 1 2 3 1 2 3 1 2 3
MYC
low high
DMSO STATTIC DMSO STATTIC
1 2 3 1 2 3 1 2 3 1 2 3
75
50
pERK
low high
DMSO STATTIC DMSO STATTIC
1 2 3 1 2 3 1 2 3 1 2 3
75
50
ERK
low high
DMSO STATTIC DMSO STATTIC
1 2 3 1 2 3 1 2 3 1 2 3
B-actin
low high
DMSO STATTIC DMSO STATTIC
1 2 3 1 2 3 1 2 3 1 2 3

Fig 4B mPKC1

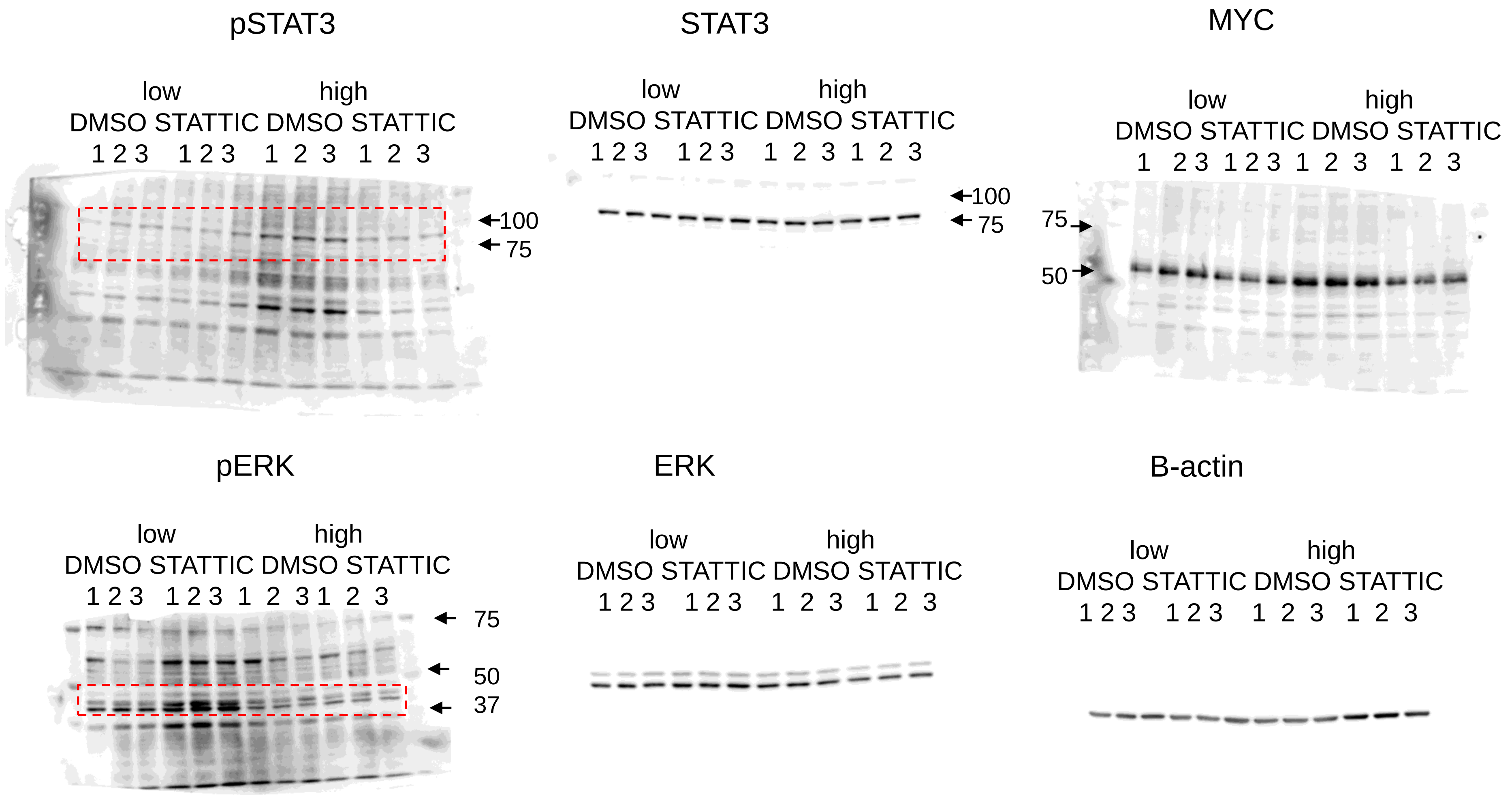
pSTAT3
low
high
DMSO STATTIC DMSO STATTIC
1 2 3 1 2 3 1 2 3 1 2 3
100
75
STAT3
low
high
DMSO STATTIC DMSO STATTIC
1 2 3 1 2 3 1 2 3 1 2 3
100
75
MYC
low
high
DMSO STATTIC DMSO STATTIC
1 2 3 1 2 3 1 2 3 1 2 3
75
50
pERK
low
high
DMSO STATTIC DMSO STATTIC
1 2 3 1 2 3 1 2 3 1 2 3
75
50
37
ERK
low
high
DMSO STATTIC DMSO STATTIC
1 2 3 1 2 3 1 2 3 1 2 3
B-actin
low
high
DMSO STATTIC DMSO STATTIC
1 2 3 1 2 3 1 2 3 1 2 3

Fig 5A PANC-1

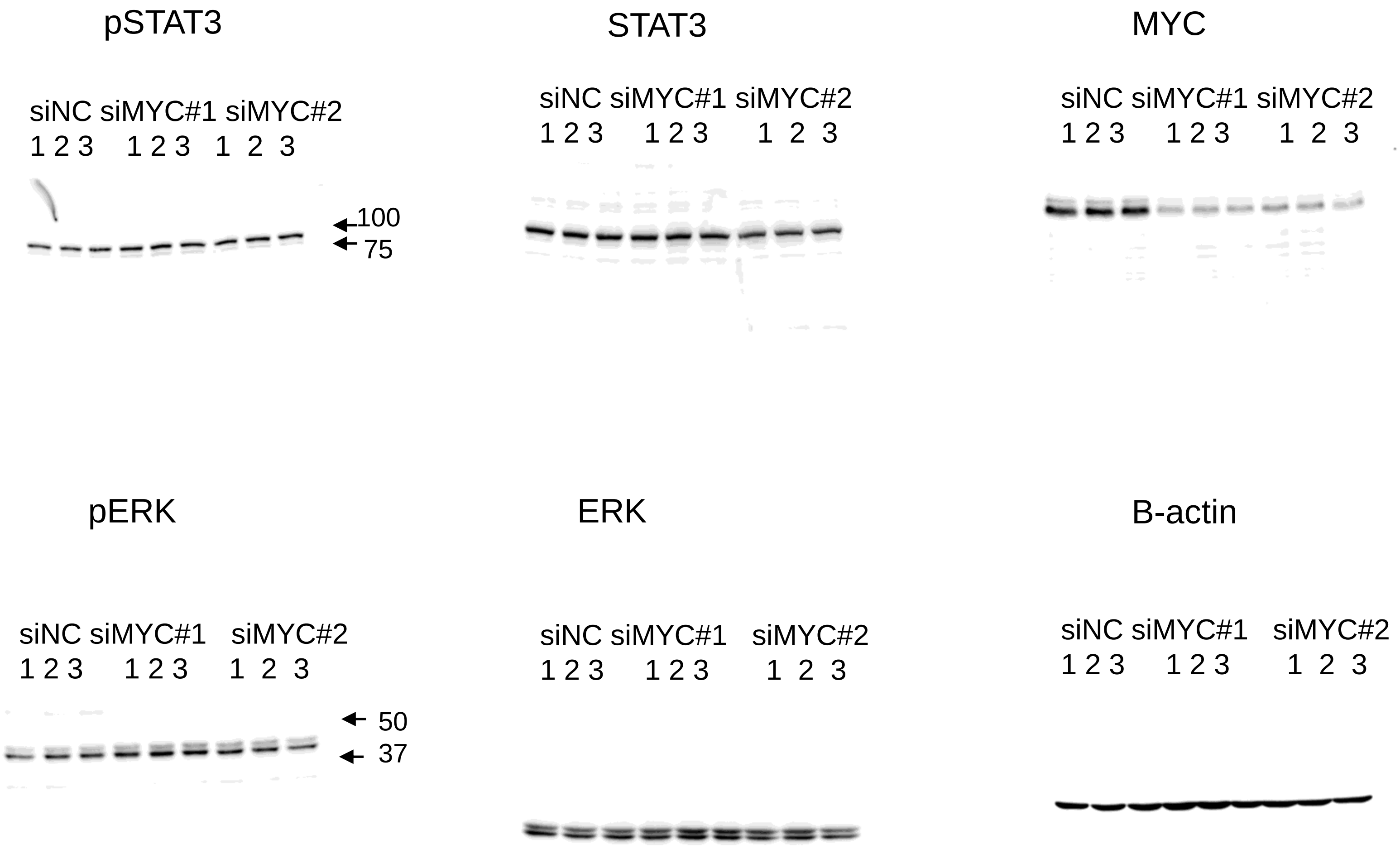
pSTAT3
siNC siMYC#1 siMYC#2
1 2 3 1 2 3 1 2 3
100
75
STAT3
siNC siMYC#1 siMYC#2
1 2 3 1 2 3 1 2 3
MYC
siNC siMYC#1 siMYC#2
1 2 3 1 2 3 1 2 3
pERK
siNC siMYC#1 siMYC#2
1 2 3 1 2 3 1 2 3
50
37
ERK
siNC siMYC#1 siMYC#2
1 2 3 1 2 3 1 2 3
B-actin
siNC siMYC#1 siMYC#2
1 2 3 1 2 3 1 2 3

Fig 5A mPKC1

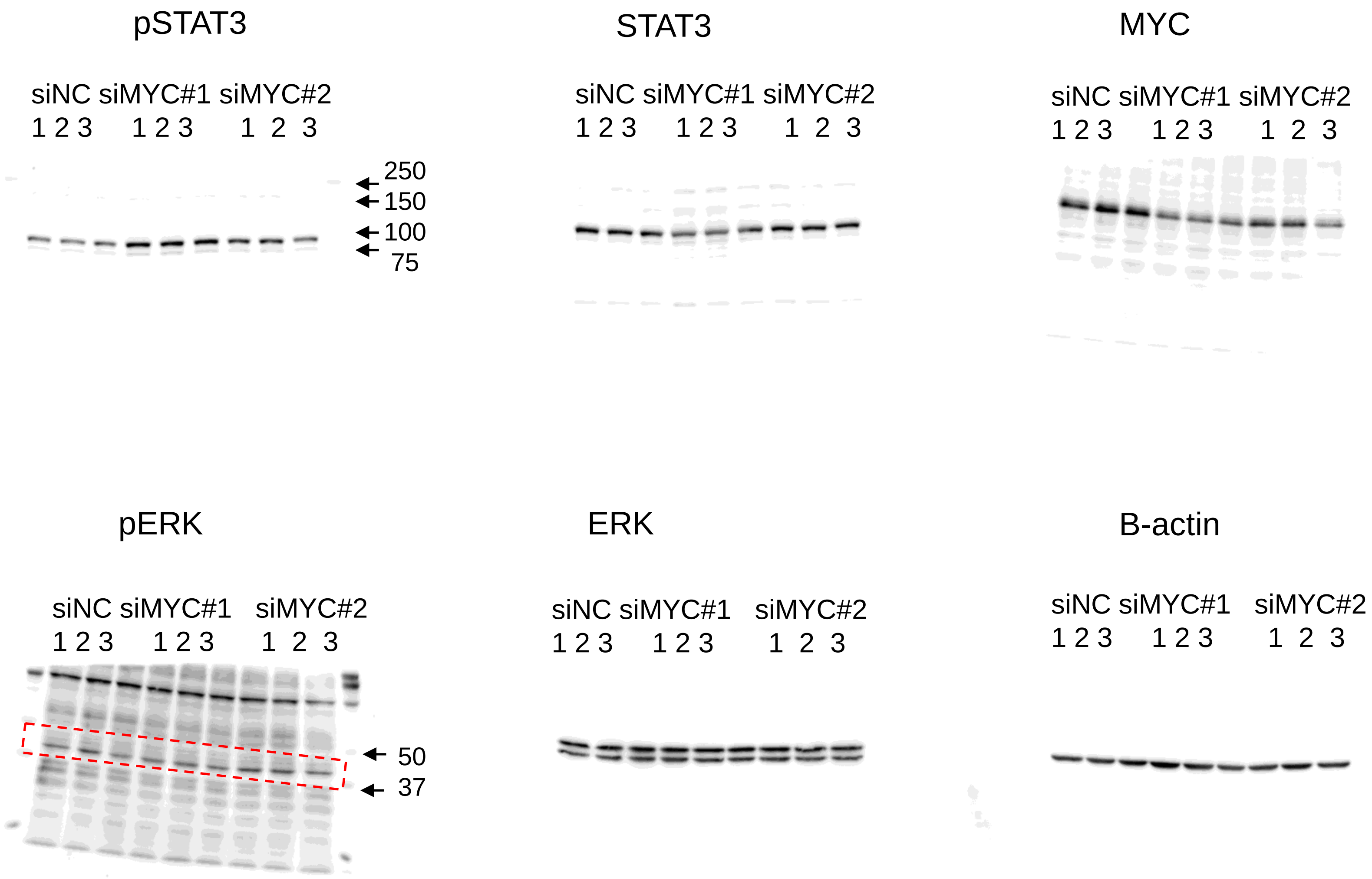

pSTAT3
siNC siMYC#1 siMYC#2
1 2 3 1 2 3 1 2 3
250
150
100
75
STAT3
siNC siMYC#1 siMYC#2
1 2 3 1 2 3 1 2 3
MYC
siNC siMYC#1 siMYC#2
1 2 3 1 2 3 1 2 3
pERK
siNC siMYC#1 siMYC#2
1 2 3 1 2 3 1 2 3
50
37
ERK
siNC siMYC#1 siMYC#2
1 2 3 1 2 3 1 2 3
B-actin
siNC siMYC#1 siMYC#2
1 2 3 1 2 3 1 2 3

S2. Fig C PANC-1 mPKC1

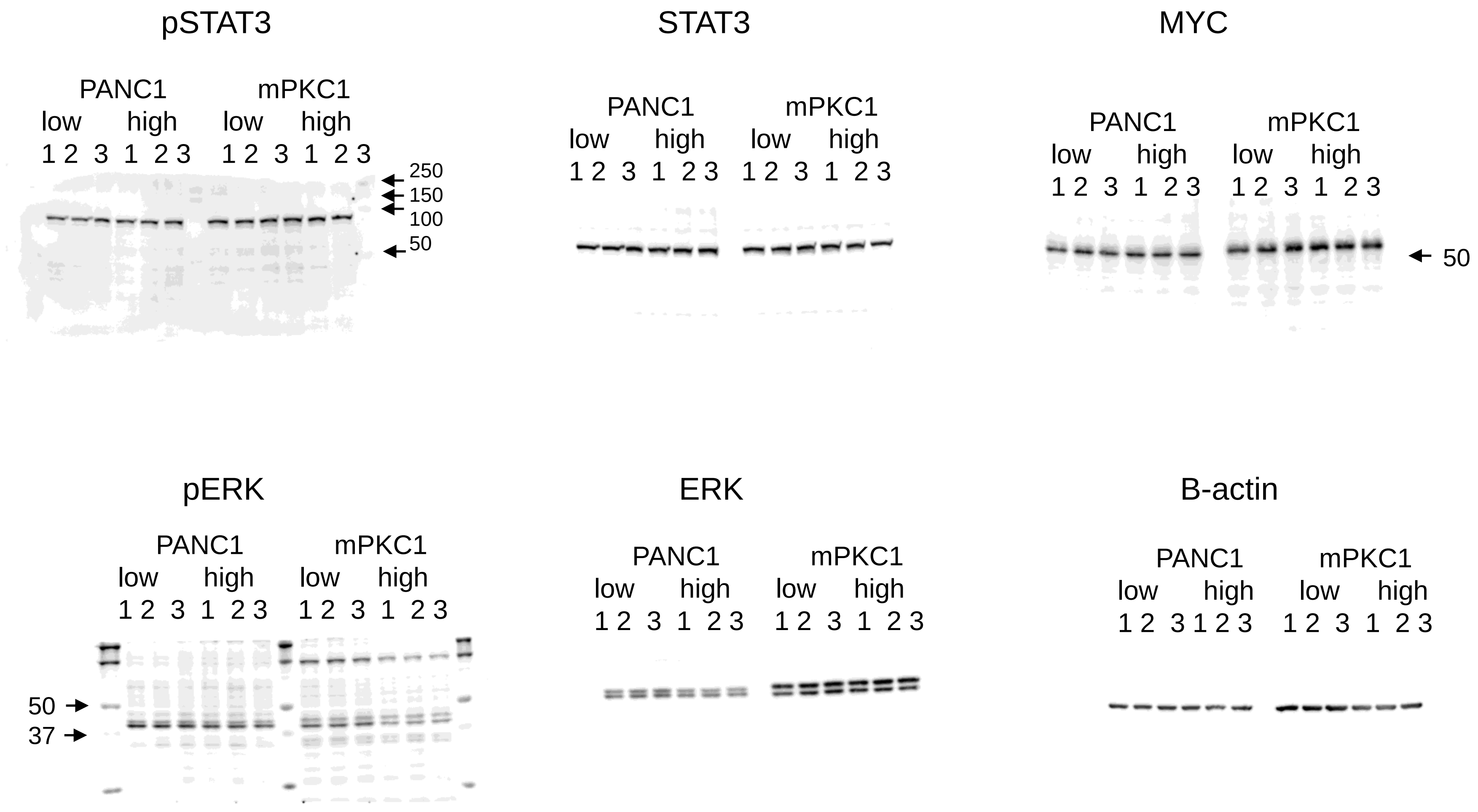

S2. Fig C BxPC3

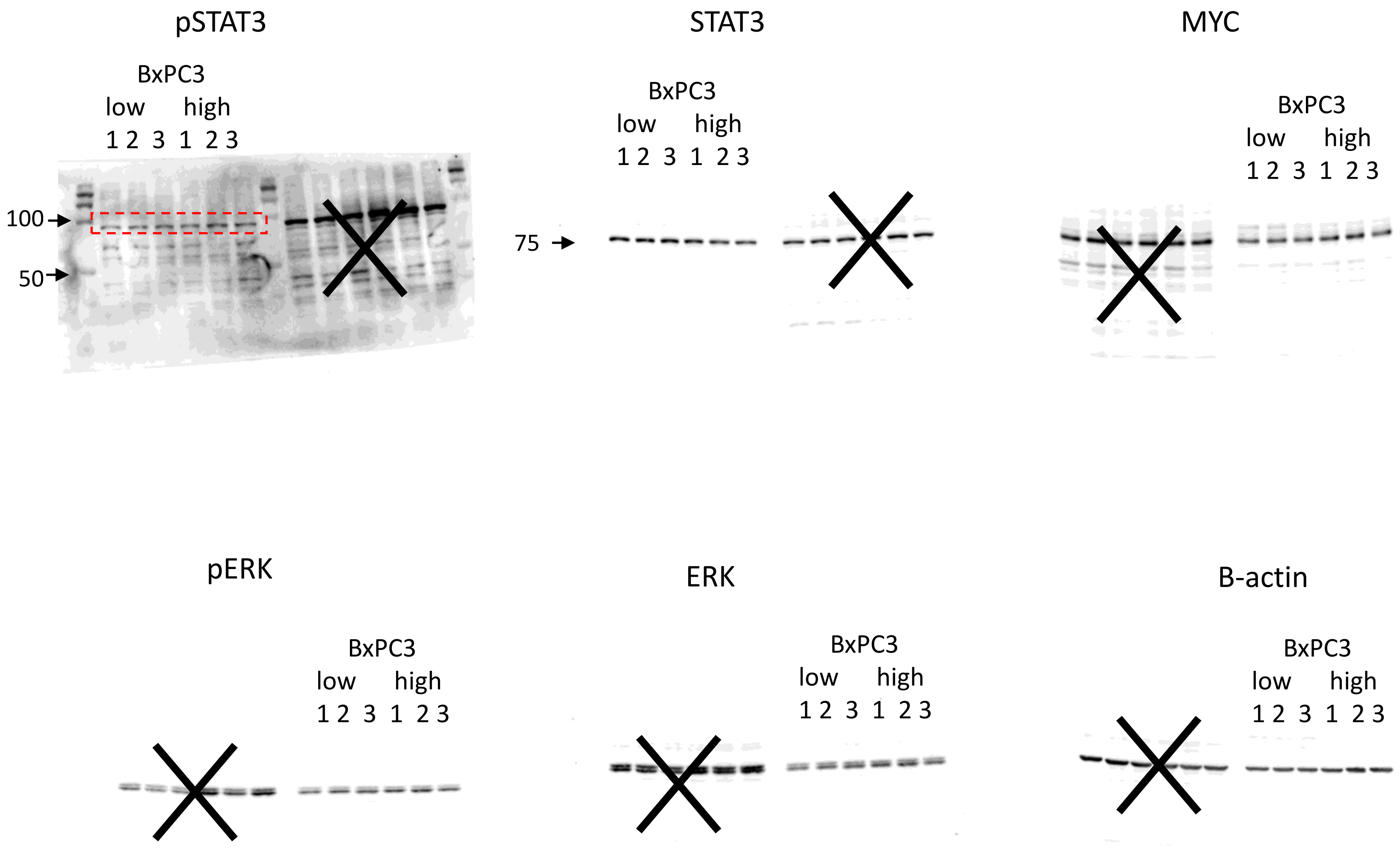

S4. Fig A PANC-1

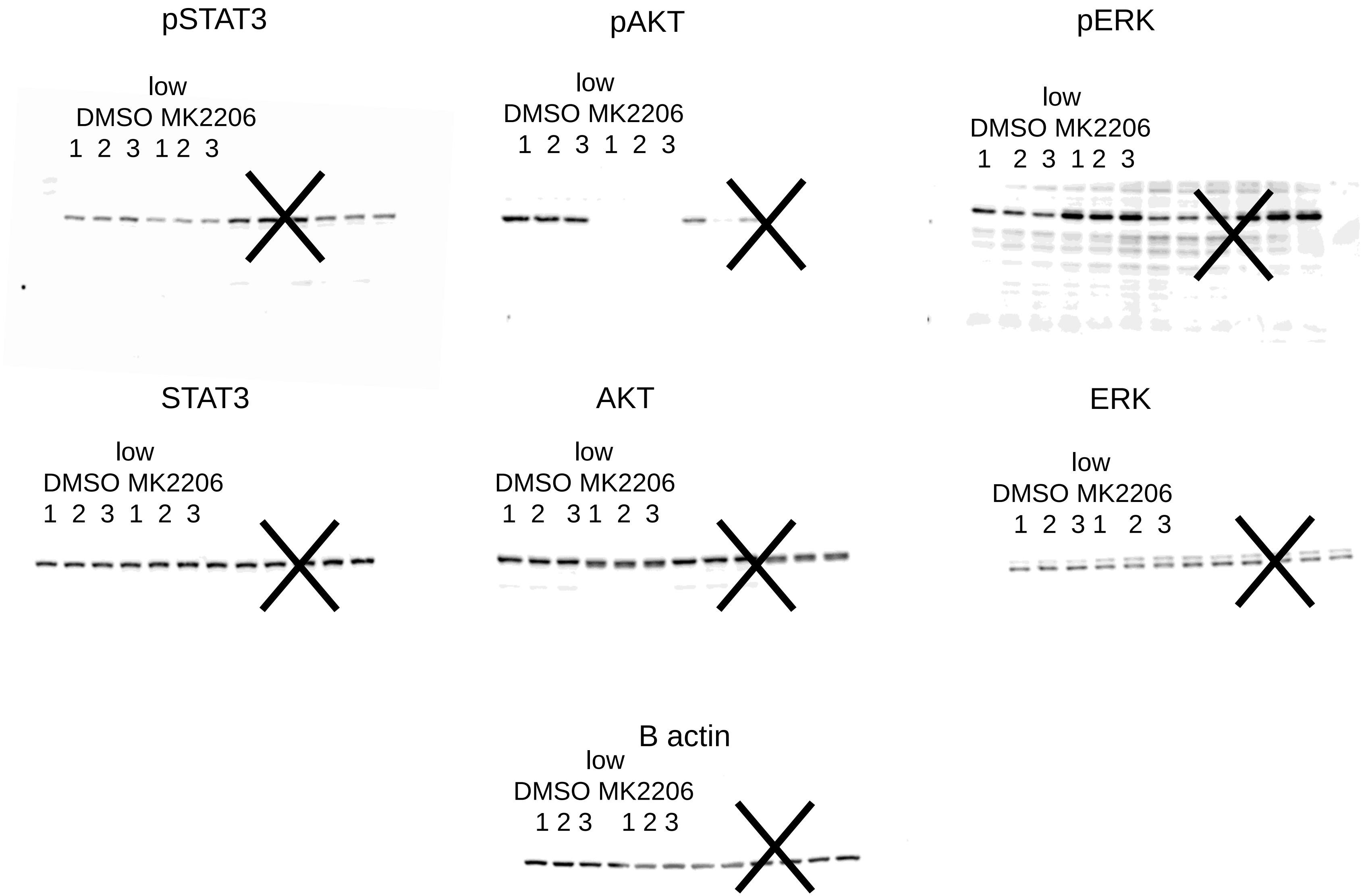

S4. Fig A mPKC1

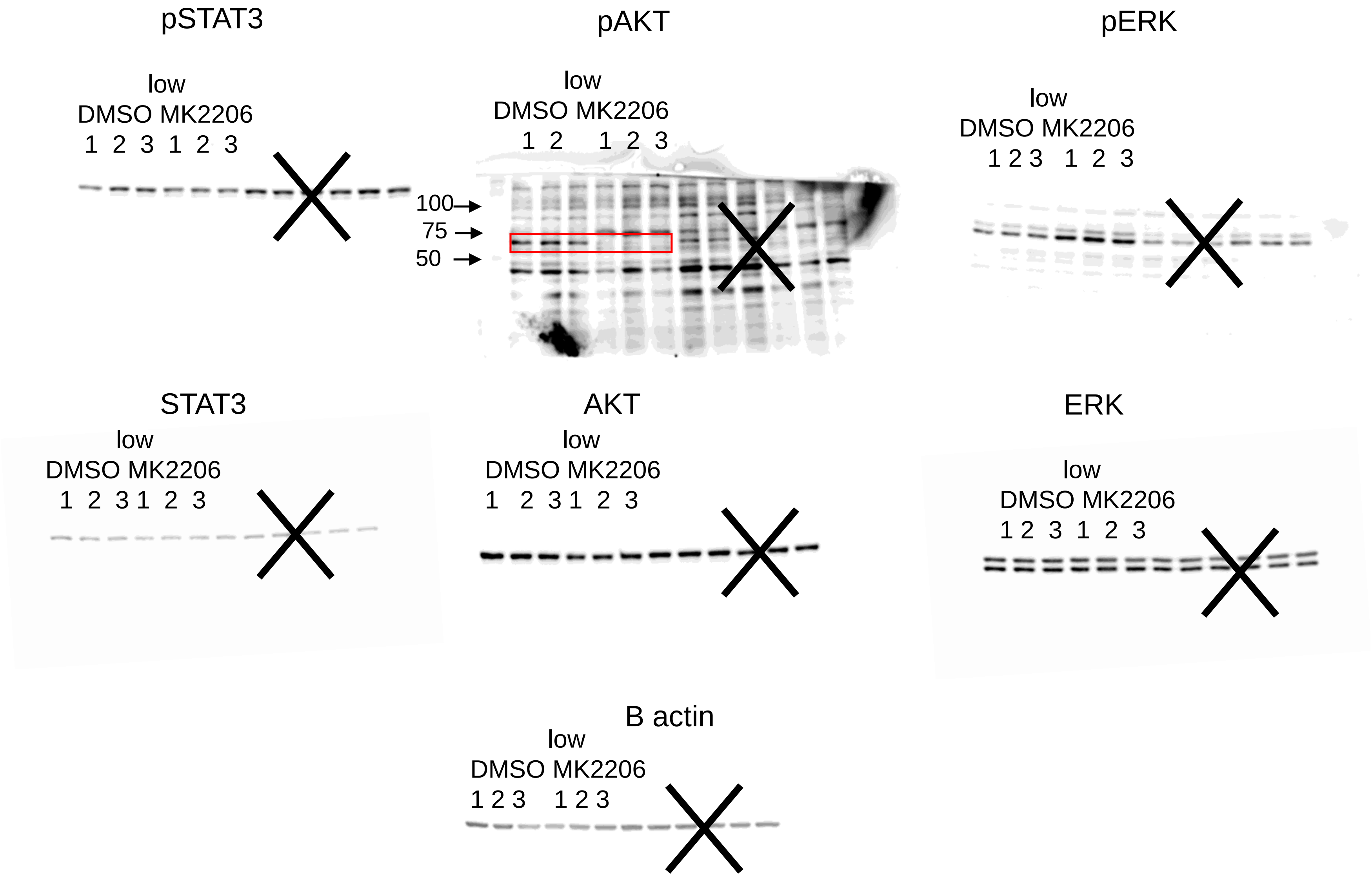

Supplement: S1 Raw images — (PDF) [file pone.0235573.s005.pdf]
